# Comparative analysis of deep Q-learning algorithms for object throwing using a robot manipulator

**DOI:** 10.3389/frobt.2025.1567211

**Published:** 2025-11-14

**Authors:** Mohammad Al Homsi, Maja Trumić, Adriano Fagiolini, Giansalvo Cirrincione

**Affiliations:** 1 Mobile and Intelligent Robots @ Panormus Laboratory (MIRPALab), Department of Engineering, University of Palermo, Palermo, Italy; 2 School of Electrical Engineering, University of Belgrade, Belgrade, Serbia; 3 Université de Picardie Jules Verne, Amiens, France

**Keywords:** artificial intelligence, deep learning, reinforcement learning, deep Q-learning, robotic manipulation, object throwing, robotics, self-attention mechanism

## Abstract

Recent advances in artificial intelligence (AI) have attracted significant attention due to AI’s ability to solve complex problems and the rapid development of learning algorithms and computational power. Among the many AI techniques, transformers stand out for their flexible architectures and high computational capacity. Unlike traditional neural networks, transformers use mechanisms such as self-attention with positional encoding, which enable them to effectively capture long-range dependencies in sequential and spatial data. This paper presents a comparison of various deep Q-learning algorithms and proposes two original techniques that use self-attention into deep Q-learning. The first technique is structured self-attention with deep Q-learning, and the second uses multi-head attention with deep Q-learning. These methods are compared with different types of deep Q-learning and other temporal techniques in uncertain tasks, such as throwing objects to unknown targets. The performance of these algorithms is evaluated in a simplified environment, where the task involves throwing a ball using a robotic arm manipulator. This setup provides a controlled scenario to analyze the algorithms’ efficiency and effectiveness in solving dynamic control problems. Additional constraints are introduced to evaluate performance under more complex conditions, such as a joint lock or the presence of obstacles like a wall near the robot or the target. The output of the algorithm includes the correct joint configurations and trajectories for throwing to unknown target positions. The use of multi-head attention has enhanced the robot’s ability to prioritize and interact with critical environmental features. The paper also includes a comparison of temporal difference algorithms to address constraints on the robot’s joints. These algorithms are capable of finding solutions within the limitations of existing hardware, enabling robots to interact intelligently and autonomously with their environment.

## 1 Introduction

In recent years, rapid developments in processor hardware have enabled artificial intelligence (AI) to significantly transform the field of robotics. AI facilitates the transition from preprogrammed automation to adaptive learning, allowing robotic systems to operate more effectively in complex and dynamic environments. In this context, the success of deep Q-learning network (DQN^1^) in mastering control policies at a human level across various Atari games (Mnih et al., 2015) has inspired many AI researchers to explore enhancements to DeepMind’s original algorithm (Hausknecht and Stone, 2015; Nair et al., 2015; van Hasselt et al., 2015). DQN has since led to significant advancements in multiple domains, particularly in robotics.

DQN is based on traditional Q-learning, which aims to determine the optimal action an agent should take in a given state to maximize cumulative rewards. However, traditional Q-learning becomes impractical in high-dimensional or complex environments, such as those often encountered in robotics. To address this, DQN uses deep neural networks (DNNs) to approximate Q-values for all states and possible actions, enabling scalable and efficient learning (Lapan, 2018). Originally introduced by researchers at DeepMind in 2013, DQN demonstrated superior performance in Atari games, surpassing human-level play through a trial-and-error learning process (Mnih et al., 2015). This success in gaming applications has encouraged researchers to apply DQN across a wide range of fields, including robotics.

In robotic applications, DQN enables robots to learn complex tasks such as navigation, manipulation, and interaction with dynamic environments (Lapan, 2018). Numerous advancements and innovations have been made within DQN, including the introduction of target networks and experience replay, both of which improve the algorithm’s stability and performance. Experience replay enhances learning efficiency by allowing the agent to learn from a diverse set of past experiences rather than relying solely on recent interactions. The use of a target network provides a stable reference for updating Q-values, helping mitigate issues related to the non-stationary nature of learning (Lapan, 2018). As research in deep reinforcement learning (DRL) for robotics continue to advance, DQN offers a promising approach for developing more intelligent and autonomous robotic systems capable of adapting to their environments and performing complex tasks with minimal human intervention.

The demand for robotic solutions in the logistics industry has increased significantly in recent years, driven by the rapid expansion of e-commerce and the challenges it presents. For example, online shopping services exert increasing pressure on logistics operations to handle packages efficiently. Although human workers possess a high degree of adaptability, they are increasingly struggling to meet the increasing demand for package handling, particularly as product volumes continue to grow sharply (Britt, 2020; Mims, 2020; Raptopoulos et al., 2020; Bombile and Billard, 2022). Robotic systems, however, offer promising adaptability to address these challenges. For instance, robotic throwing can provide a substantial advantage in scenarios where speed and precision are critical as it extends a robot’s effective working range beyond its physical and hardware limitations (Frank et al., 2006). This capability is particularly valuable in robotic pick-and-place tasks, as demonstrated in autonomous bi-manual robots such as Delta robots, which have proven especially effective in applications like waste sorting (Raptopoulos et al., 2020; Hassan et al., 2022).

**TABLE 1:** List of acronyms.

Acronym	Meaning
AI	Artificial intelligence
ML	Machine learning
NN	Neural network
RL	Reinforcement learning
DQN	Deep Q-learning
DNN	Deep neural network
DRL	Deep reinforcement learning
DH parameters	Denavit–Hartenberg parameters
PPO	Proximal policy optimization
SAC	Soft actor-critic
SARSA	State–action–reward–state–action
DQN-N	Deep Q-learning with noisy network
DDQN	Double deep Q-learning
PER-DQN	Prioritized experience replay deep Q-learning
DDQN-N	Double deep Q-learning with noisy networks
PER-DDQN	Prioritized experience replay double deep Q-learning
DQN-SA	Structured self-attention-based deep Q-learning
DQN-MHA	Deep Q-network with multi-head attention
DDQN-SA	Structured self-attention-based double deep Q-learning
PER-DDQN-SA	Prioritized experience replay and structured self-attention-based double deep Q-learning
TAR	Total average return
DM	Dynamic model
DDPG	Deep deterministic policy gradient

Controlling the trajectory of an object using a robotic system is a complex task; however, it is essential for performing tasks in dynamic environments. In such scenarios, the robot must continuously adapt and adjust the position and velocity of the end-effector to ensure the ball lands accurately in the basket. Two main challenges arise under these conditions. First, the robot must compute a valid solution or determine the correct trajectory configuration to successfully throw the ball into the target. Second, it must accurately adapt its throwing parameters in real time, including the release position and velocity, to overcome environmental constraints (Bombile and Billard, 2023).

Controlling the trajectory of an object with a robotic system seems a complex task, however, it is essential when dealing with tasks in dynamic environments. In these scenarios, the robot must continuously adapt and adjust its parameters such as the end-effector’s position to ensure the ball lands accurately in the basket position. There are two main challenges in these scenarios. First, the robot must determine a solution or the correct configuration for the robot’s trajectory movement to throw the ball into the target. Second, it must accurately adapt its throwing parameters–including release position, joint values, and direction in real-time to overcome constraints in the environment (7).

### 1.1 Related work

The challenge of dynamic object manipulation has received increasing attention in recent years. The problem of robotic catching is often framed as an interception task, similar to traditional robotic catching scenarios (Burridge et al., 1995; Lynch and Mason, 1999; Schill and Buss, 2018; Dong et al., 2020; Satici et al., 2022). While catching involves intercepting an object moving toward the robot, throwing requires greater control over the robot’s trajectory. In this case, the trajectory depends on several factors, including the initial and final positions of the joints, their velocities, and environmental constraints.

Robotic throwing (Mason and Lynch, 1993; Raptopoulos et al., 2020; Bombile and Billard, 2022) offers significant advantages in terms of time and energy efficiency compared to static pick-and-place approaches. In such cases, advanced vision systems are employed to track and guide thrown objects with high precision (Frank et al., 2006). Other researchers have used hybrid learning and optimization methods to determine the parameters required for accurate object throwing (Bombile and Billard, 2023).

Various robotic platforms have been used for object throwing, including 1-DoF and 2-DoF systems (Frank et al., 2006; Mason and Lynch, 1993), industrial robots (Raptopoulos et al., 2020; August et al., 2010; Zhang et al., 2012), and humanoid robots (Kim et al., 2008; Satici et al., 2016). For instance, the KUKA KR-16 robot has been shown to throw objects to targets 2.5 m away (August et al., 2010). The UR5 robot has been adapted to throw objects of different shapes and sizes (Zeng et al., 2020). More recently, the Franka Emika Panda robot demonstrated adaptive throwing capabilities, adjusting its behavior in response to dynamic conditions (Liu et al., 2022). These developments reflect the growing interest and potential of robotic throwing applications using deep learning models, laying the groundwork for future innovations across various industries.

Artificial intelligence enhances the performance of robotic systems but still requires retraining to adapt to sudden environmental changes or newly introduced constraints in robotic manipulators. When new manipulators are deployed, updated datasets are often necessary for retraining. However, current algorithms face several challenges in adapting to such changes. First, many adaptation algorithms rely heavily on human expertise to identify and address constraints, often requiring carefully planned strategies. Undetected constraints or malfunctions can cause the robot’s task to fail. Moreover, some AI algorithms cannot compensate for constraints in real time.

Researchers have explored various approaches to improve robotic adaptability and mitigate these limitations. For instance, neural networks have been used to estimate new workspaces for robotic arms with locked joints, although these methods often fall short in providing real-time compensation (Sivasamy et al., 2019). Other research uses acoustic filtering to identify constraints using sound sensors (Hu et al., 2019). Deep learning algorithms have also been applied to improve fault diagnosis in robotic systems under harsh conditions, with a primarily focus on fault identification (Zhang et al., 2019; Costa et al., 2019). These algorithms have been primarily used for fault identification, leaving a research gap regarding their performing under such conditions.

In the context of robotic throwing, researchers have explored ways to significantly enhance robot capabilities by enabling them to throw objects into a moving basket while avoiding obstacles—offering an advantage over manual object placement using algorithms such as soft actor-critic (SAC) and deep deterministic policy gradient (DDPG) (Kasaei and Kasaei, 2024). Other studies have combined several AI models, including deep convolutional encoder–decoder architectures for image segmentation, stochastic neural networks for physics simulation, and reinforcement learning (Zeng et al., 2020).

In one approach, a reinforcement learning agent is used to generate forward-phase actuation, while a dynamic model (DM) predicts the landing position. Both the agent and the DM are implemented as neural networks with a single hidden layer (Bianchi et al., 2023). It could be observed that the robot’s dynamics significantly influence the learning process. Other AI approaches include the use of dual neural networks to predict ping-pong ball trajectories (Lin et al., 2020) and more advanced architectures such as autoencoders (Gonzalez, 2020).

Previous studies have highlighted key challenges in this domain, related to limited training data, prediction errors, and insufficient accuracy in throwing tasks. Moreover, there is a lack of comprehensive analysis addressing scenarios where the robot encounters both environmental constraints and hardware failures during the throwing process.

### 1.2 Contributions

This paper compares the performance of various DQN algorithms applied to the task of object throwing using an articulated serial robot manipulator. In particular, one of the paper’s contributions is two novel algorithms that integrate attention mechanisms into the DQN framework. The effectiveness of these attention-based algorithms is evaluated against standard DQN algorithms, with and without attention layers, through a detailed performance analysis. By incorporating attention mechanisms into DQN, the robot manipulator’s ability to capture relevant information is improved, enabling more efficient decision-making during the throwing process.

This research further tackles real-world limitations such as hardware faults (e.g., joint restrictions) and environmental obstacles (e.g., nearby walls or target obstacles), demonstrating how these challenges can be effectively addressed through reward shaping within the Q-learning framework. To the best of our knowledge, this is the first study to evaluate the performance of learning algorithms for robotic throwing under such conditions, as summarized in Table 2.

**TABLE 2:** Positioning of proposed research within the related work of throwing an object using a robot manipulator.

Paper	Approach	Environmental constraint/hardware failure	Learning model
Zeng et al. (2020)	Deep learning residual physics	Neither is considered	Convolutional residual network
August et al. (2010)	Ballistic-trajectory-based motion planning. An illustrative result	Neither is considered	Not ML-based
Liu et al. (2022)	Adaptive throwing using trajectory planning. The approach requires accurate trajectory tracking	The robot is disturbed using an external interaction force	ML is used to build the object’s inverted flying dynamics
Kim et al. (2008) and Satici et al. (2016)	Feasibility of humanoid throwing. The focus is on physical execution, not learning	Neither is considered	Not used
Bianchi et al. (2023)	Throwing an object using a soft manipulator	Neither is considered	NN + RL
Kasaei and Kasaei (2024)	Throwing an object with obstacle avoidance	There is an obstacle near the target.	SAC and DDPG
This paper	Deep Q-learning approaches	We consider three cases: 1) obstacle near the target, blocking the ball’s trajectory; 2) obstacle near the robot, constraining its motion; and 3) failure in the robot’s joint	DQN, Noisy DQN, DDQN, PER, and SA variants

Finally, an extensive comparative analysis is presented, examining the performance of standard and attention-augmented DQN variants across a range of constrained and unconstrained scenarios, highlighting their potential for improved performance in unstructured environments.

## 2 Methods and solution

To solve throwing or pick-and-place tasks using Q-learning and deep reinforcement learning algorithms, it is necessary to define the action and state spaces the algorithm operates in, along with several key design decisions, as outlined below:

1. Actions: The actions correspond to joint adjustments, which vary across experiments. Each adjustment value is computed as a function of the difference between the basket’s center position and the predicted landing point.
2. States: The states include the starting state, working state, and end state. An additional modification has been introduced: every new position of the end-effector corresponds to a new state.
3. Reward function: The reward is based on the error distance, defined as the distance between the basket’s center and the landing point from the most recent throw.
4. Denavit–Hartenberg (DH) parameters: These parameters are defined for each of the robots used.

### 2.1 Reinforcement learning approach

An agent is defined as an entity that interacts with the environment by performing actions, collecting information (observations or states), and receiving rewards (positive or negative) (Lapan, 2018). In reinforcement learning, there are two main types of actions an agent can perform:

- Policy-based actions: The agent learns a policy, which is a mapping of states to actions. This policy can be stochastic—where action–state pairs are based on probabilities for different actions, as observed in algorithms such as DQN during exploration or proximal policy optimization (PPO)—or deterministic, where each state maps to a single specific action (Lapan, 2018).
- Value-based actions: Here, the agent estimates the value of each action–state pair and selects actions based on these values (e.g., by choosing the action with the maximum value). For example, in Q-learning, the Q-function estimates the expected reward in a Q-table, and the agent selects the action with the highest Q-value (Lapan, 2018; Van Hasselt et al., 2016; Sutton and Barto, 2018).

The epsilon-greedy strategy in Q-learning is used to encourage exploration of the environment. When the agent encounters a new state with unknown Q-values, it needs to explore different actions to estimate their rewards since there is no prior knowledge. This strategy prevents the agent from sticking to a suboptimal policy and ensures it does not miss better actions or paths (Van Hasselt et al., 2016; Sutton and Barto, 2018). In contrast, algorithms such as PPO or SAC perform exploration automatically. Because these algorithms initialize network weights randomly during training, the output initially follows a uniform probability distribution, resulting in random agent behavior (Lapan, 2018).

A DRL policy is the decision-making mechanism in RL that guides the agent’s actions based on observations from the environment. Traditional temporal difference algorithms such as Q-learning, state–action–reward–state–action (SARSA), and expected SARSA define policies by estimating state values mapped to each action, selecting the action with the highest expected return (Lapan, 2018). When the environment has a small, discrete action set, Q-learning can efficiently approximate the value of each state–action pair and select the best action.

However, when the action set is large, directly calculating Q-values for every action becomes inefficient. In such cases, neural networks (NNs) are used to approximate Q-values (Lapan, 2018). In DQNs, the NN outputs the expected reward values for actions given a specific state, represented as scalar values.

**FIGURE 1:**
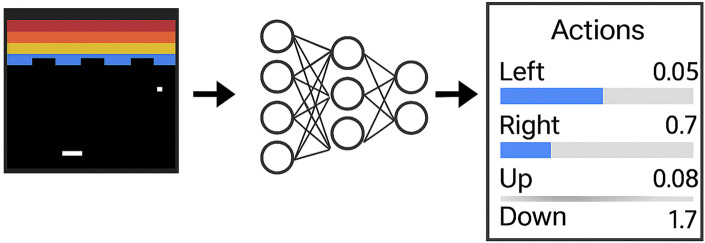
Neural network-based policy approximation for a discrete action space (adapted from Lapan (2018)).

There are multiple ways to implement this output:

- The NN outputs identifiers for all possible actions in an array (representing a discrete set of actions). Although this is a simple approach, it may not be the most effective way to handle discrete action sets (Lapan, 2018).
- The NN outputs a probability distribution over the agent’s actions, as illustrated in Figure 1 (Lapan, 2018).

The pieces of information or knowledge collected by the agent from the environment at a specific time and state are called the RL state. The state captures various aspects, including the agent’s location, the surrounding environment, and sometimes information on previous states, actions taken, and rewards received.

The activities performed by the agent in DRL are called actions. Actions can be discrete, continuous, or a combination of both. Discrete actions represent fixed behaviors, such as moving left or right in a grid, moving up or down, or pressing and releasing buttons. Continuous actions specify variable values related to the agent, such as the angle position of a steering wheel or the angular velocity of robot joints. Some environments require multiple simultaneous actions, like adjusting an angle by a discrete increment while setting a continuous angular speed (Lapan, 2018). In this paper, the actions in DQN or Q-learning are described as varying between experiments. Importantly, the joint positions in all experiments respect the physical constraints and limitations of the robot arm.

The RL reward is a scalar value obtained from the environment that indicates the degree to which the agent’s previous action was beneficial or detrimental. Rewards can be positive or negative and large or small. The timing of reward delivery depends on the experiment: rewards can be given continuously at every interaction or only once during the agent’s lifetime (Lapan, 2018). When rewards are sparse—given only once—other reward signals are 0 until the final reward is received. The reward reflects the success or failure of the agent’s previous actions. However, receiving a high reward for certain actions does not guarantee the absence of negative consequences from earlier decisions. For example, a risky policy might yield a high immediate reward but lead to poor outcomes overall (Lapan, 2018).

### 2.2 Q-learning approach

Q-learning is a model-free, off-policy algorithm that uses a lookup table to learn the optimal action-value function (Q-function) for a given state. In the context of throwing balls using a robot arm, the Q-function represents the expected reward for taking a particular action (e.g., adjusting the arm’s angle or the throwing velocity) in a specific state (e.g., the current position of the ball or the robot arm). However, learning the optimal policy through Q-learning requires extensive trial-and-error exploration, which can be time-consuming and inefficient (Van Hasselt et al., 2016).

Additionally, Q-learning may struggle with continuous action spaces, posing limitations for tasks such as robotic ball throwing that demand precise control over continuous variables (Sutton and Barto, 2018). Q-learning is classified as an off-policy algorithm because it learns and improves a policy that is different from the policy currently being executed by the agent. This contrasts with on-policy algorithms, in which the agent learns and improves the policy it is actively following (Van Hasselt et al., 2016).

#### 2.2.1 Q-learning choice of hyperparameters

**FIGURE 2:**
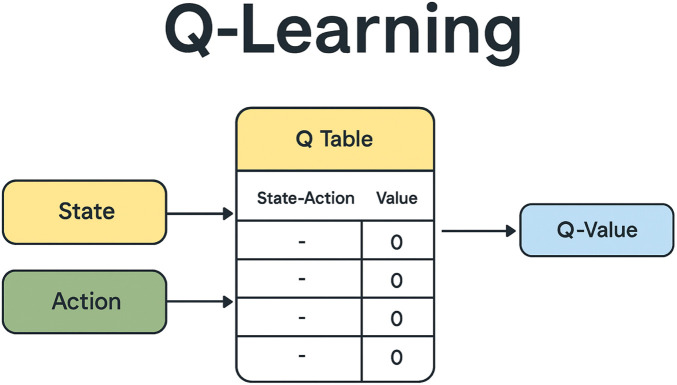
Tabular representation of state–action values (Q-table) in the Q-learning algorithm.

The Q-learning algorithm aims to find the optimal policy—a sequence of actions—that maximizes the expected cumulative reward over time. This is achieved by updating Q-values using the Bellman equation, which relates the Q-value of a current state–action pair to the Q-values of the next state–action pairs, as illustrated in Figure 2.

The Q-function, or Q-learning update rule, is expressed using the Bellman equation as follows:

$$Q\left(s,a\right)⟵Q\left(s,a\right)+\alpha \left[r+\gamma \underset{a^{\prime }}{\max}Q\left(s^{\prime },a^{\prime }\right)-Q\left(s,a\right)\right].$$

-
α is the learning rate, a value between 0 and 1 that determines how much new information overrides old estimates. A higher α places more weight on recent rewards.
-
Q(s,a) is the estimated cumulative reward for taking action a in state s .
-
r is the immediate reward received after executing action a in state s .
-
γ is the discount factor, also between 0 and 1, which determines the importance of future rewards compared to immediate rewards.
-
$\max_{a^{\prime }}Q\left(s^{\prime },a^{\prime }\right)$ represents the maximum Q-value over all possible actions $a^{\prime }$ in the next state $s^{\prime }$ . Q-learning selects actions by considering both the immediate and the maximum future reward.
-
ϵ represents the exploration–exploitation trade-off. A higher ϵ encourages exploration (trying new actions), while a lower ϵ favors exploitation (choosing the best-known action). A decaying ϵ schedule is commonly used, starting with high exploration that decreases over time (Sutton and Barto, 2018; Van Hasselt et al., 2016).

This approach allows the agent to first explore its environment through random actions and then use the gained experience to select the most appropriate actions for making an optimal policy (Sutton and Barto, 2018; Watkins, 1989; Mnih et al., 2013; Mnih et al., 2015; Van Hasselt et al., 2016). The Q-learning update rule is applied iteratively as the agent interacts with the environment, enabling it to learn the optimal policy for choosing actions across different states to maximize cumulative rewards over time. SARSA, an on-policy reinforcement learning algorithm, differs from Q-learning by replacing the term $\max_{a^{\prime }}Q\left(s^{\prime },a^{\prime }\right)$ with $Q\left(s^{\prime },a^{\prime }\right)$ in the update rule:

$$Q\left(s,a\right)⟵Q\left(s,a\right)+\alpha \left[r+\gamma Q\left(s^{\prime },a^{\prime }\right)-Q\left(s,a\right)\right].$$

The agent in the SARSA algorithm updates its Q-values based on the actions it actually takes. This characteristic indicates that the learned policy is tightly coupled with the exploration strategy used during training. As a result, SARSA often yields more conservative behavior, making it more robust to the agent’s exploration decisions. In contrast, expected SARSA is an off-policy reinforcement learning algorithm that improves upon standard SARSA. Instead of relying on the single action taken by the agent, it uses the expected value over all possible next actions in the policy’s distribution. This provides a smoother and often more stable learning process. The update rule for expected SARSA is provided as follows:

$$\begin{array}{cc} Q\left(s_{t},a_{t}\right) & \leftarrow Q\left(s_{t},a_{t}\right)\\ & +\alpha \left[r_{t+1}+\gamma E_{a_{t+1}∼\pi }\left[Q\left(s_{t+1},a_{t+1}\right)\right]-Q\left(s_{t},a_{t}\right)\right]. \end{array}$$

Here,

-
$Q\left(s_{t},a_{t}\right)$ is the current estimate of the action-value function for state $s_{t}$ and action $a_{t}$ .
-
α is the learning rate, determining the step size of the Q-value update.
-
$r_{t+1}$ is the reward received after taking action $a_{t}$ in state $s_{t}$ .
-
γ is the discount factor, controlling the weight of future rewards relative to immediate rewards.
-
$E_{a_{t+1}∼\pi }\left[Q\left(s_{t+1},a_{t+1}\right)\right]$ denotes the expected value of $Q\left(s_{t+1},a_{t+1}\right)$ over all possible actions $a_{t+1}$ that may be taken in the next state $s_{t+1}$ under policy π .

This approach aims to reduce variance and improve both learning efficiency and stability. One of the key hyperparameters in Q-learning is the exploration–exploitation strategy. As is well known, this strategy balances two competing objectives: selecting the best-known action based on current knowledge (exploitation) and exploring new actions that might yield better long-term rewards (exploration) (Wikipedia, 2023; AI-ML, 2023).

The immediate reward R(s,a) is designed to encourage actions that move the throwing result closer to the basket. It is defined as follows:

$$R=1-0.5\cdot \left(d_{t+1}-d_{t}\right)+\left\{\begin{array}{cc} -20,\; & \text{if }d_{t+1}\geq d_{t}\\ +0.3,\; & \text{if }d_{t+1}<d_{t}, \end{array}\right.$$

where $d_{t}$ is the distance– i.e., the straight-line distance from the landing point to the center of the basket in throw t , and $d_{t+1}$ is the corresponding distance in the next throw.

In experiments, an additional reward of +0.3 is added if $d_{t+1}<d_{t}$ . Conversely, a penalty of −20 is imposed if the error increases. This adjustment helps the agent distinguish between joints with constraints and those without. For instance, if a joint with constraints is repeatedly selected, its Q-value will decrease due to poor performance, making it less likely to be chosen in future iterations. A numerical example illustrating this behavior is provided later in the paper.

**FIGURE 3:**
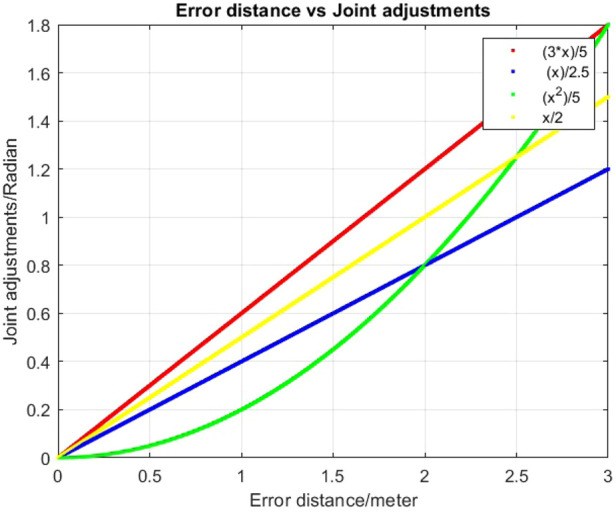
Different functional mappings from distance $d_{t}$ to joint adjustments in Q-learning.

Moreover, Q-learning easily allows mapping the joint adjustment values to the error distance $d_{t}$ . When $d_{t}$ is large, the algorithm tends to apply larger joint adjustments. Conversely, when $d_{t}$ is small, finer adjustments are made to refine the trajectory (Figure 3). Although adjusting the joint weights may influence the results, the same weight is consistently used across all experiments.

#### 2.2.2 Q-learning states and actions

A *Q-learning state* encompasses the information available to an agent about its environment at a specific moment in time. This includes various aspects such as the agent’s current location, surrounding objects, and the history of previous states, actions taken, and rewards received. As illustrated in Figure 2, the Q-learning algorithm relies on a Q-table, typically represented as an array of Q -values where each row corresponds to a state and each column corresponds to an action. The generic entry $a_{i,j}$ in the Q-table represents the value $Q\left(s_{i},a_{j}\right)$ (Sutton and Barto, 2018). At the start of the algorithm, all entries in the Q-table are initialized to 0. The agent begins to explore the environment, and the Q-function iteratively updates these values based on interactions. Over time, this iterative process yields improved approximations of the optimal Q-values.

The typical Q-learning update loop is as follows:

1. Parameters: step size a in (0, 1], ϵ> 0
2. Initialize Q(s,a) , for all s∈S and a∈A(s) , arbitrarily except Q(terminal,⋅)=0

3. Loop for each episode:
  - Initialize s
  - Loop for each step of the episode:
    1. Choose a from s using policy derived from Q (e.g., ϵ -greedy).
    2. Take action a and observe R and ${\text{s}}^{\prime }$ .
    3. Calculate the new reward using the Q-function.
    4. s = $s^{\prime }$

  - until s is terminal

The ϵ -greedy policy is defined as follows (Li, 2023):

$$\pi \left(a|s\right)=\left\{\begin{array}{cc} \frac{\epsilon }{\left|A\left(s\right)\right|}+\left(1-\epsilon \right)\; & \text{if }a=\arg\max_{a^{\prime }}Q\left(s,a^{\prime }\right)\\ \frac{\epsilon }{\left|A\left(s\right)\right|}\; & \text{otherwise}. \end{array}\right.$$

Here,

-
π(a|s) is the probability of taking action a in state s .
-
ϵ is the exploration rate (a small positive constant, typically between 0 and 1).
-
|A(s)| is the number of possible actions in state s .
-
Q(s,a) is the action-value function.

In this paper, the initial state is defined as the starting configuration of the system, while the ending state corresponds to a configuration in which Q-learning identifies a successful throwing solution. A key modification introduced in this work is the assignment of a unique state to every new position of the end-effector. This results in a significantly larger number of working states compared to previous implementations that used a single state representation, thereby enhancing performance.

A comparison is conducted between two scenarios: (i) Q-learning with a limited number of discrete states and (ii) Q-learning with an expanded state space that includes a greater number of end-effector positions. This comparison is illustrated in Figures 15–17. By using a finer state discretization, the algorithm is less reliant on pure exploitation. This is important as excessive exploitation can cause the algorithm to overlook alternative promising trajectories. The broader state space increases the diversity of solutions available for the throwing task, often allowing the algorithm to converge more quickly. However, this also introduces a risk of over-exploration, where the agent continually explores new actions without sufficiently exploiting learned policies. To mitigate this issue, the Q-learning algorithm is further modified: if an action results in a negative reward, the agent returns to the previous state. This simple yet effective modification ensures consistent convergence by discouraging the repetition of poor actions and reinforcing successful behaviors.

**FIGURE 4:**
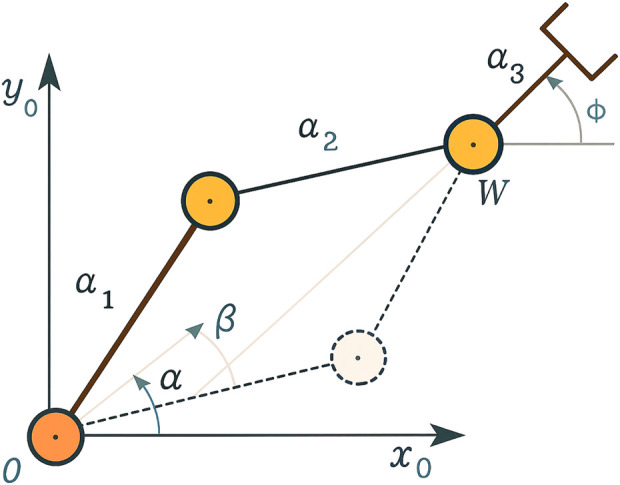
Admissible configuration of a two-link planar arm (Siciliano, 2008).

**FIGURE 5:**
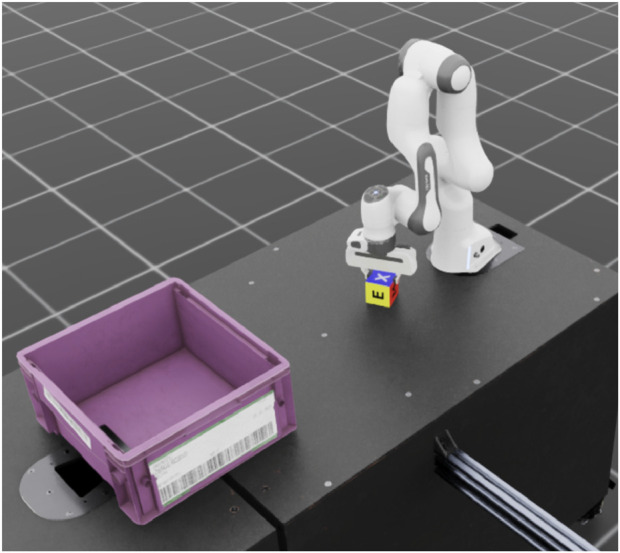
Franka Emika Panda robot whose simulated model is used in the robotic throwing task (Albu-Schäffer et al., 2016).

The actions in Q-learning are defined as joint adjustments, and they vary depending on the experiment, as described below:

1. For the PhantomX Pincher Robot Manipulator (Figure 10, DH parameters provided in Table 3), used in pick-and-place tasks, the defined actions for each joint are as follows:
  - Increasing or decreasing the joint angle $\theta_{k}$ by w times the adjustment value, where k∈{1,2,3,4} and w∈{1,2,3} .
2. For the two-link planar arm (Figure 4, DH parameters provided in Table 4), used in the throwing task, the defined actions for both joints are as follows:
  - Increasing or decreasing the joint angle $\theta_{k}$ by a fixed adjustment value, where k∈{1,2} .
  - Increasing or decreasing the throwing angle by 0.1.
3. For the Franka Emika Panda robot (Figure 5, DH parameters provided in Table 5), also used in the throwing task, the defined actions are as follows:
  - Increasing or decreasing the joint angle $\theta_{k}$ by a fixed adjustment value, where k∈{1,2,3,4,5,6,7} .
  - Increasing or decreasing the throwing angle by 0.1.

**TABLE 3:** DH parameters for PhantomX Pincher robot (standard convention).

Joint i	$\theta_{i}$ (variable)	$d_{i}$ (m)	$a_{i}$ (m)	$\alpha_{i}$ (rad)
1	$\theta_{1}$	0.05	0	π/2
2	$\theta_{2}$	0	0.105	0
3	$\theta_{3}$	0	0.105	0
4	$\theta_{4}$	0	0.075	0

**TABLE 4:** DH parameters for a two-link planar robot (standard convention).

Joint i	$\theta_{i}$ (variable)	$d_{i}$ (m)	$a_{i}$ (m)	$\alpha_{i}$ (rad)
1	$\theta_{1}$	0	$L_{1}$	0
2	$\theta_{2}$	0	$L_{2}$	0

**TABLE 5:** DH parameters for Franka Emika Panda (standard convention).

Joint i	$\theta_{i}$ (variable)	$d_{i}$ (m)	$a_{i}$ (m)	$\alpha_{i}$ (rad)
1	$\theta_{1}$	0.333	0	0
2	$\theta_{2}$	0	0	−π/2
3	$\theta_{3}$	0.316	0	π/2
4	$\theta_{4}$	0	0.0825	π/2
5	$\theta_{5}$	0.384	−0.0825	−π/2
6	$\theta_{6}$	0	0	π/2
7	$\theta_{7}$	0.107	0	0

The adjustment value is computed based on the Euclidean distance between the position of the target (basket) center and the landing point of the most recent throw as follows:

$$\text{Adj\_value}=w_{1}\;d_{t},$$

where $d_{t}$ denotes the distance between the two points and $w_{1}$ is a scaling factor. The updated joint values are constrained to remain within their predefined ranges and must satisfy all joint-specific constraints.

#### 2.2.3 Constraint compensation using Q-learning

Q-learning is employed to update the joint positions, resulting in new coordinates for the end-effector. These coordinates are calculated using the DH parameters, which describe the kinematics of the robotic arm. Various approaches exist for computing the correct end-effector coordinates required for the throwing task. Once these coordinates are determined, inverse kinematics (IK) can be applied to derive the corresponding joint values. Techniques such as the Jacobian inverse method and numerical optimization are commonly used to solve IK problems. These methods account for the arm’s specific kinematic structure and any joint faults or constraints (Group, 2024; Learning, 2025).

However, traditional IK solutions are often slow and struggle to handle multiple joint failures. The Q-learning algorithm overcomes these limitations by adapting to environmental changes and predefined policies. The Q-function is shaped to assign higher rewards to actions that result in throws closer to the basket. If an action violates a joint constraint, the associated reward is reduced, guiding the algorithm to select alternative actions involving unconstrained joints in subsequent iterations. Thus, Q-learning provides a more adaptive and fault-tolerant solution for dynamically updating joint positions during the throwing task.

### 2.3 Approaches using DQNs

**FIGURE 6:**
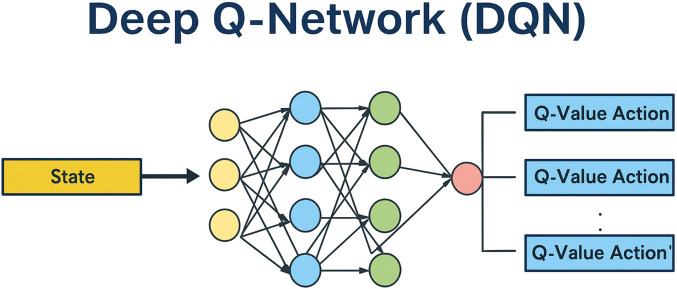
Structure of the deep Q-network, illustrating the flow from input state representation to Q-value outputs for each discrete action.

Deep reinforcement learning combines neural networks with RL techniques to address high-dimensional decision-making problems. This integration enables interactive learning in complex and dynamic environments. A key reason behind the growing adoption of deep RL is its demonstrated effectiveness across diverse applications and its compatibility with modern computational platforms (Figure 6). Several types of DQNs have been developed to enhance performance in various settings (Lapan, 2018; Sorokin et al., 2015). These include standard DQN, double DQN, N-step DQN, noisy networks, and prioritized experience replay (PER) DQN, along with attention-based extensions.

This paper explores several advanced DQN variants:

- Noisy networks (DQN-N): they enhance exploration efficiency by injecting noise into the network weights.
- Double DQNs (DDQNs): they mitigate overestimation by decoupling action selection and evaluation through two separate networks and improving stability and accuracy—especially useful in tasks requiring precision, such as robotic throwing.
- Prioritized replay DQNs (PER-DQNs): they increase learning efficiency by prioritizing more informative experiences during training.
- Noisy double DQNs (DDQN-Ns): they combine the benefits of DQN-Ns and DDQNs for robust exploration and stable learning.
- PER with double DQNs (PER-DDQNs): they integrate the strengths of PER-DQNs and DDQNs to enhance both sampling efficiency and Q-value estimation.
- Self-attention-based DQNs (DQN-SA, DQN-MHA): they incorporate attention mechanisms to help the model focus on critical input features, improving learning in high-dimensional environments such as throwing tasks.
- Structured self-attention double DQNs (DDQN-SA): they combine DDQNs with structured self-attention to further refine decision making.
- PER-DDQN-SA: it integrates PER, DDQN, and self-attention to leverage the advantages of all three approaches.

Recently, deep reinforcement learning studies have investigated the robustness of policies. A robust policy is desirable because it should not be sensitive to random seeds or hyperparameters. In some cases, such as when a validation environment is unavailable, off-policy evaluation can be used to estimate policy performance using only pre-collected data. This method allows RL agents to predict the effectiveness of new policies without deploying them in the real world (Li, 2023).

In this paper, the following metrics are used to evaluate the performance of different DQN algorithms:

- Policy performance: the total average return (TAR) is the most common measure of policy performance (Li, 2023):

$$\text{TAR}=E_{s_{0}∼d_{s}}\left[\frac{1}{N}\overset{N}{\underset{i=1}{\sum }}R_{i}\right],$$

where

-
TAR is the total average return,
-
$E_{s_{0}∼d_{s}}$ is the expectation over initial states $s_{0}$ sampled from distribution $d_{s}$ ,
-
N is the number of episodes or samples, and
-
$R_{i}$ is the return in the i th episode.

This equation expresses the expected average return computed over N episodes, with the initial states drawn from a given distribution.

- Learning speed: this refers to the rate at which an RL agent improves its performance over time through training and interactions with the environment (Li, 2023).
- Learning accuracy: this metric assesses how closely a learned policy or value function approximates the optimal one (Li, 2023).

#### 2.3.1 DQN with self-attention

This novel approach applies two different self-attention mechanisms within DQN. Self-attention mechanisms selectively focus on the most relevant parts of the input data, enhancing the neural network’s capacity to process large inputs.

**FIGURE 7:**
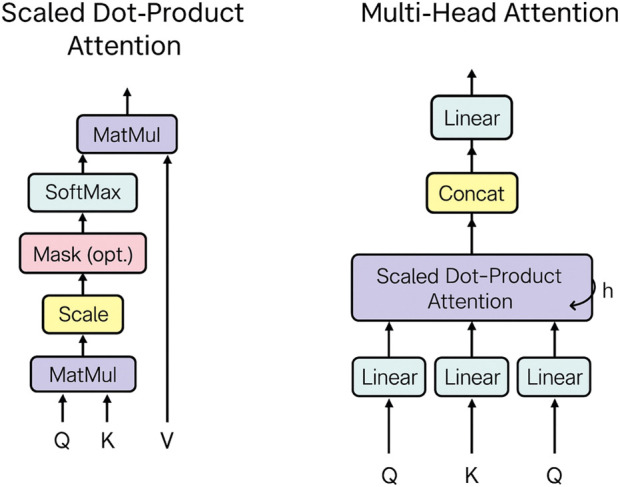
Visualization of scaled dot-product attention (left) and multi-head attention (right) (Vaswani et al., 2017).

The key concepts of attention mechanisms are as follows:

- Self-attention computes relationships between different parts of the input to generate context-aware representations. It maps a query and a set of key-value pairs to an output, as shown in Figure 7. The output is a weighted sum of the values, where weights are computed using a compatibility function. This function measures the alignment between the query and each corresponding key (Vaswani et al., 2017).
- Scaled dot-product attention calculates attention scores by taking the dot products of queries and keys, scaling by $\sqrt{d_{k}}$ , and applying a softmax function to obtain attention weights (Vaswani et al., 2017). The inputs are queries and keys (each of dimension $d_{k}$ ) and values (dimension $d_{v}$ ), arranged in matrices Q , K , and V , respectively (Figure 7). The attention output is computed as follows (Ba et al., 2016):

$$Attention\left(Q,K,V\right)=softmax\left(\frac{QK^{⊤}}{\sqrt{d_{k}}}\right)V.$$

- Multi-head attention uses multiple attention heads to capture different aspects of relationships within the data (Figure 7).

Incorporating self-attention mechanisms into DQNs enhances the agent’s ability to focus on the most relevant features. This leads to improved decision-making and increased learning efficiency. Self-attention can be integrated into a DQN as follows:

- The representation of the state uses attention mechanisms to process and encode state information. For example, the state vector in a throwing task with a robot manipulator is processed using self-attention to extract crucial features. This allows the network to prioritize the most significant parts of the input data.
- Self-attention-based DQNs incorporate self-attention layers within the network architecture. In this paper, self-attention layers are added to the network except for the final fully connected layers. This modification significantly improves the network’s ability to handle complex state representations and enhances learning performance.

Integrating attention mechanisms into DQN improves the identification and prioritization of critical information from input data. By focusing on important features, the learning process becomes more efficient, reducing the number of episodes required to achieve good performance and accelerating training. Moreover, attention mechanisms enhance the agent’s ability to generalize. This facilitates better performance in unseen or complex environments and allows the agent to adapt more effectively to new scenarios and challenges.

#### 2.3.2 Multi-head attention in DQNs

**FIGURE 8:**
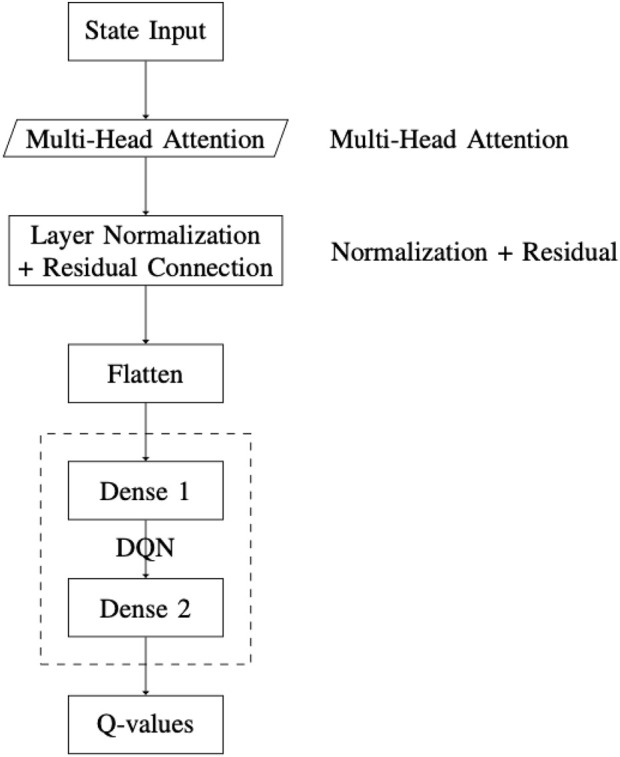
Overview of the DQN-MHA architecture, which integrates multi-head attention into the deep Q-network framework.

Integrating multi-head attention into a DQN can enhance the agent’s ability to learn more complex policies. This improvement is achieved by enabling the agent to simultaneously focus on multiple aspects of the state representation. The architecture of the DQN with multi-head attention (DQN-MHA) used in this paper is shown in Figure 8 and includes the following components.

- A state input layer that receives a sequence representing the environment’s current and previous states as a vector, illustrated in Figure 8. The input sequence consists of the latest eight states.
- An attention layer that applies a self-attention mechanism to the input state, allowing the model to focus on the most relevant parts. Each attention head processes the same input sequence but from a different perspective. The outputs of the heads are concatenated to form the multi-head attention output. This approach mimics the [query, value] format by passing the same input twice. Following Keras (2023), the value is also used as a key. In this work, 12 attention heads are employed (Vaswani et al., 2017).
- Normalization and residual layers are applied to the multi-head attention output. The residual connection allows the input to bypass sub-layers and be added directly to the output. Layer normalization is applied after the residual connection to stabilize learning and improve convergence. The normalization layer uses $\epsilon =1\times 10^{-6}$ . Its output type is a KerasTensor with shape (1,No.Neurons) .
- A flatten layer reshapes the multi-dimensional tensor (1,No.Neurons) into a one-dimensional tensor of the same shape. This facilitates the transition from attention layers to fully connected dense layers, without altering the data values or structure.
- A DQN consisting of fully connected dense layers applies linear transformations followed by ReLU activations. This enables the learning of complex patterns from the integrated attention outputs.
- A final dense output layer produces q-values for each possible action. These Q-values estimate the expected future rewards for actions given the current state and are used for decision-making in reinforcement learning.

Within the DQN-MHA model, each attention head receives the same input sequence, processes it independently using distinct (q,k) weight matrices, and generates an output that reflects a unique perspective. This design implements horizontal self-attention, enabling the model to extract diverse features from the same input, which enhances information abstraction.

The output of each attention head i is defined as follows:

$${\text{Attention}}_{i}\left(S\right)=\text{softmax}\left(\frac{SW_{i}^{Q}{\left(SW_{i}^{K}\right)}^{⊤}}{\sqrt{d_{k}}}\right)\left(SW_{i}^{V}\right).$$

The outputs from all heads are concatenated and projected:

$$\text{MHA}\left(S\right)=\text{Concat}\left({\text{Attention}}_{1}\left(S\right),\ldots ,{\text{Attention}}_{h}\left(S\right)\right)W^{O}.$$

A residual connection followed by layer normalization is applied:

$$Z_{2}=\text{LayerNorm}\left(S+\text{MHA}\left(S\right)\right).$$

The output is then flattened and passed through two fully connected layers with ReLU activations:

$$z=\text{Flatten}\left(Z_{2}\right),$$

$$h_{1}=\text{ReLU}\left(W_{1}z+b_{1}\right),$$

$$h_{2}=\text{ReLU}\left(W_{2}h_{1}+b_{2}\right).$$

The final Q-value is computed as follows:

$$Q\left(s_{t},a\right)=W_{3}h_{2}+b_{3}.$$

Notations:

-
$s_{t}\in R^{n}$ : current state vector
-
$S=\left[s_{t-7},\ldots ,s_{t}\right]\in R^{8\times n}$ : sequence of the last eight states
-
Q(s,a) : Q-value for state s and action a

-
A : number of actions
-
h : number of attention heads
-
$d_{k}$ , $d_{v}$ : dimensions of keys/queries and values
-
$W_{i}^{Q},W_{i}^{K},W_{i}^{V}$ : weight matrices for query, key, and value in head i

-
$W^{O}$ : output projection matrix for the multi-head attention
-
$Z_{2}$ : output after residual connection and LayerNorm
-
z : flattened vector of $Z_{2}$

-
$W_{1},W_{2},W_{3}$ : weight matrices for the fully connected layers
-
$b_{1},b_{2},b_{3}$ : bias terms for the fully connected layers
-
$h_{1},h_{2}$ : hidden layer outputs in the feedforward network

#### 2.3.3 Structured self-attention in DQNs

**FIGURE 9:**
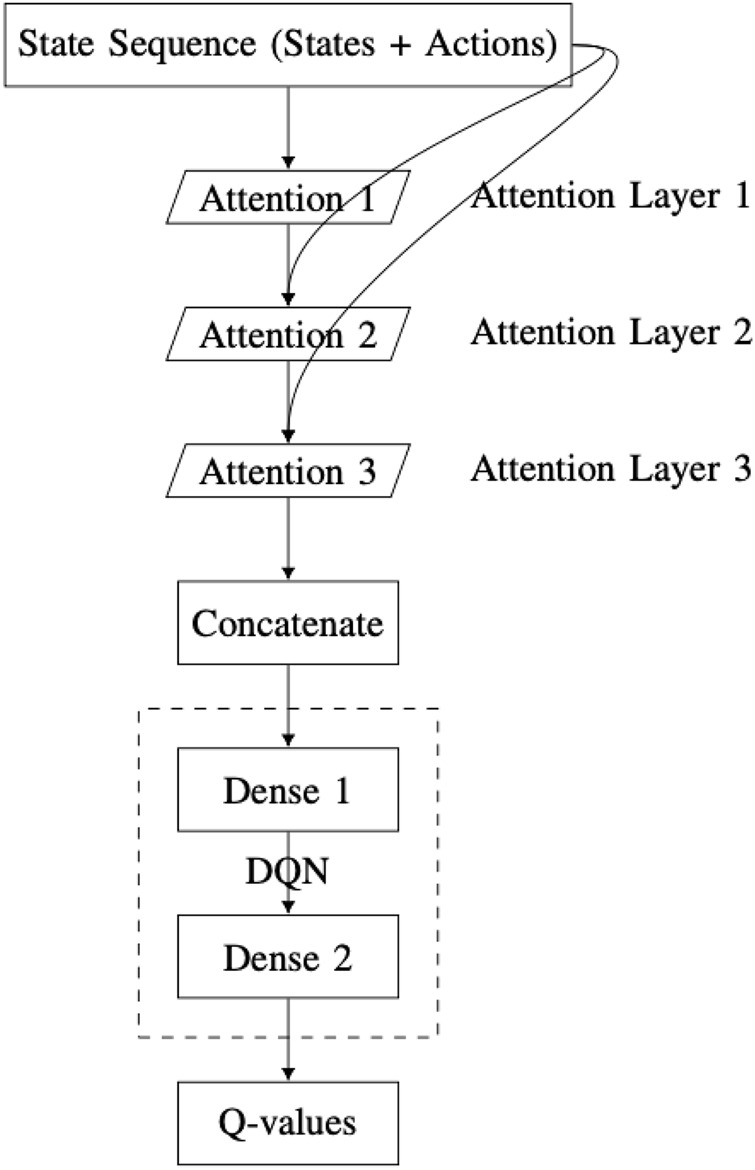
Overview of the DQN-SA architecture integrating self-attention layers within the deep Q-network.

Integrating scaled dot-product attention with a DQN can improve the agent’s ability to learn more complex policies. This is achieved by enabling the agent to focus on different aspects of the state representation simultaneously. The DQN-SA architecture proposed in this work (Figure 9) consists of the following components:

- State input layer: it receives a sequence of states, identical to the input used in the DQN with MHA architecture.
- Self-attention layer 1: it applies a scaled dot-product attention mechanism to the input sequence, offering a first perspective (“point of view”) on the state.
- Self-attention layer 2: it processes the original state input again, now incorporating the output of the first self-attention layer.
- Self-attention layer 3: it takes the result from the second layer and combines it with the first layer’s output to form a refined third-level perspective.
- Concatenation layer: it merges the outputs of all three self-attention layers into a single vector. This integration step allows the network to combine multiple attention-derived representations of the state.
- DQN module: it comprises a series of fully connected dense layers, identical to the architecture used in the MHA-based DQN, which processes the concatenated output.
- Q-values output layer: It is a final dense layer that computes the Q-values for each possible action.

This architecture processes the input state through successive self-attention layers, with each layer forming its own representation based on the output of the previous layer. As a result, the network creates a vertical self-attention hierarchy. This layered structure enables the model to extract increasingly abstract features during training. However, there is a risk of losing certain low-level details. To mitigate this, outputs from all attention layers are concatenated, ensuring that essential information is retained.

For DQN-SA, let $Z_{0}=S$ . For each attention layer l=1,2,3 ,

$$Q_{l}=Z_{l-1}W_{l}^{Q},\;K_{l}=Z_{l-1}W_{l}^{K},\;V_{l}=Z_{l-1}W_{l}^{V},$$

$${\text{Attention}}_{l}=\text{softmax}\left(\frac{Q_{l}K_{l}^{⊤}}{\sqrt{d_{k}}}\right)V_{l},$$

$$Z_{l}={\text{Attention}}_{l}.$$

After computing the outputs of the attention layers, they are concatenated and passed through a fully connected feedforward network:

$$Z_{\text{concat}}=\text{Concat}\left(Z_{1},Z_{2},Z_{3}\right),\;z=\text{Flatten}\left(Z_{\text{concat}}\right),$$

$$h_{1}=\text{ReLU}\left(W_{1}z+b_{1}\right),\;h_{2}=\text{ReLU}\left(W_{2}h_{1}+b_{2}\right),$$

$$Q\left(s_{t},a\right)=W_{3}h_{2}+b_{3},$$

where

-
$S\in R^{n}$ : input state sequence vector.
-
$Z_{0}=S$ : initial input to the first attention layer.
-
$W_{l}^{Q},W_{l}^{K},W_{l}^{V}$ : query, key, and value weight matrices for layer l , respectively.
-
$d_{k}$ : dimensionality of the keys and queries.
-
$Q_{l},K_{l},V_{l}$ : transformed input matrices in layer l .
-
${\text{Attention}}_{l}$ : output of the scaled dot-product attention for layer l .
-
$Z_{l}$ : output of attention layer l .
-
$Z_{\text{concat}}$ : concatenated outputs from all attention layers.
-
z : flattened version of $Z_{\text{concat}}$ .
-
$W_{1},W_{2},W_{3}$ : weight matrices of the dense layers.
-
$b_{1},b_{2},b_{3}$ : bias vectors for the dense layers.
-
$h_{1},h_{2}$ : intermediate activations from the fully connected layers.
-
$Q\left(s_{t},a\right)$ : predicted Q-value for taking action a in state $s_{t}$ .

#### 2.3.4 Comparison analysis of DQNs based on self-attention

The primary distinction between the two architectures lies in the direction of self-attention: DQN-SA employs vertical self-attention, while DQN-MHA uses horizontal self-attention. In DQN-MHA, multiple attention heads operate in parallel on the same input but produce different outputs due to variations in their query–key weight matrices. Each head captures a distinct perspective of the input, enabling the network to extract diverse features relevant for solving deep reinforcement learning problems.

In contrast, DQN-SA generates each layer’s output based on the preceding self-attention layer, forming a hierarchical abstraction of the input. This vertical stacking enhances the model’s capacity for abstraction but may lead to a loss of finer details. To mitigate this, the input vector of each self-attention layer is fused with the original state input. Although DQN-SA shares structural similarities with DQN-MHA, it functions in a sequential (vertical) manner. Considering the complexity of the throwing task, additional self-attention layers or multi-head attention in DQN-SA may not be necessary.

## 3 Result

This section describes the application of the above techniques to different robots performing various tasks when limited by physical boundaries or affected by failures. These scenarios are addressed using different reinforcement learning algorithms. The corresponding code can be found on GitHub^2^.

### 3.1 Q-learning for robot throwing

#### 3.1.1 Q-learning for pick-and-place tasks

**FIGURE 10:**
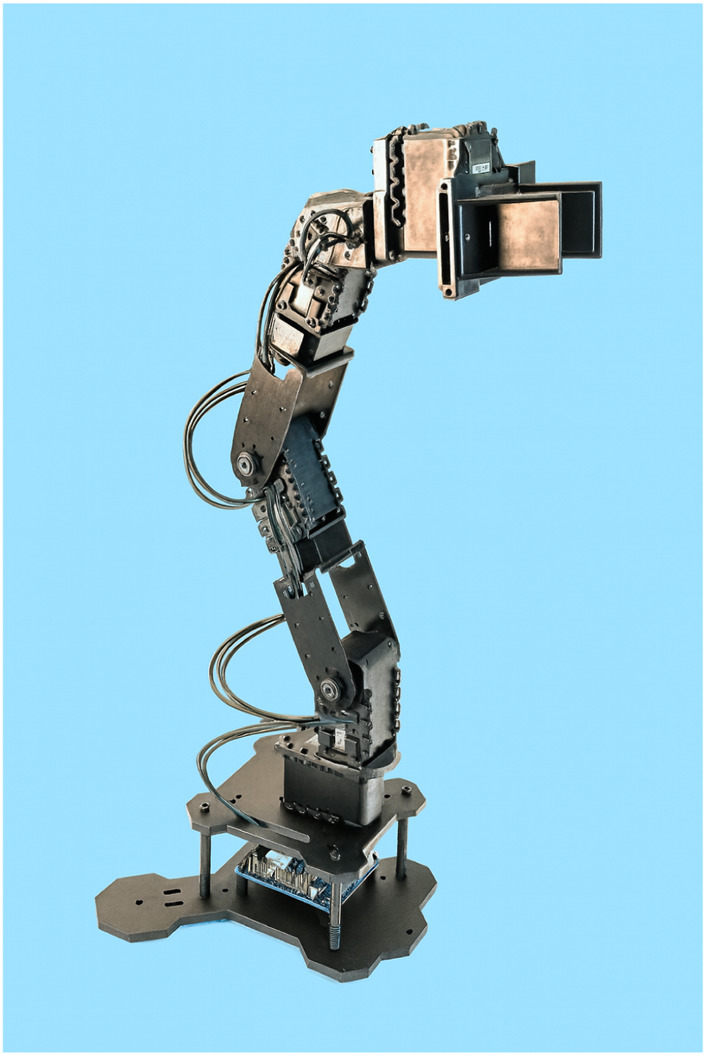
PhantomX Pincher robotic manipulator.

To evaluate the performance of the Q-learning algorithm in the presence of physical constraints (e.g., hardware failures or environmental boundaries), two experiments were conducted for pick-and-place tasks (Wiki, 2024). The robot used is the PhantomX Pincher, shown in Figure 10, with its DH parameters provided in Table 3.

- Experiment #1: The robot is tasked with moving from an initial position to point A (0.1698,0.0272,−0.2081) and subsequently to point B (0.2313,−0.0401,−0.1333) in Cartesian space. After reaching point A, two simultaneous failures occur: one restricts the motion of the second joint, while the other completely locks the fourth joint. Despite these failures, the Q-learning algorithm is able to adapt and find alternative solutions, avoiding reliance on the faulty joints. The robot reaches near point A with a position error of 0.8778 cm and near point B with an error of 0.3817 cm. The total execution time is 10.25 s.
- Experiment #2: To assess the algorithm’s behavior under multiple joint failures, the robot must sequentially reach three target points in Cartesian space: A=(0.1698,0.0272,−0.2081) , B=(0.2313,−0.0401,−0.1333) , and C=(−0.0436,0.2610,−0.182) . After reaching point A, a failure affects the first joint, limiting its motion. Upon reaching point B, two additional failures occur: the second joint becomes fully blocked, and the third joint’s range is severely restricted. The Q-learning algorithm successfully guides the robot to all three points despite the joint limitations, achieving position errors of 0.7176 cm, 0.7921 cm, and 0.9853 cm for points A, B, and C, respectively. The total time taken is 18.83 s.

#### 3.1.2 Constraint compensation using reward function

Reward function design, also known as reward function engineering, is among the most challenging aspects of reinforcement learning, particularly when addressing robotic constraints such as joint range limits and torque saturation. To ensure that the agent operates within its physical limitations, penalty terms can be introduced into the reward function, e.g., for actions that violate joint constraints. Properly tuning these penalty weights not only encourages physically feasible behavior but also accelerates the learning process by guiding the agent toward safer and more effective solutions.

Although numerous types of constraints are relevant in robotic systems, we focus now solely on total joint failures. The reward function is modified accordingly to enable efficient task execution while respecting these constraints.

$$R=1-0.5\cdot \left(d_{t+1}-d_{t}\right)+\left\{\begin{array}{cc} -20,\; & \text{if }d_{t+1}\geq d_{t}\\ +0.3,\; & \text{if }d_{t+1}<d_{t} \end{array}\right..$$

The above reward function incorporates a distance-based shaping term and a discrete bonus/penalty mechanism. The additional reward values ( +0.3 and −20 ) were selected empirically through systematic experimentation, evaluating various alternatives to identify those that produced optimal learning performance and stable agent behavior.

The reward is computed based on the distance, defined as the Euclidean distance between the landing point and the center of the basket. The center of the basket is taken as the coordinate origin (0,0). A positive bonus of +0.3 is applied whenever the distance decreases, whereas a large negative penalty of −20 is applied when the distance increases or remains the same as in the previous attempt. This design encourages the agent to perform actions that improve task success while simultaneously adhering to joint constraints.

- Analysis without sudden constrains.

**FIGURE 11:**
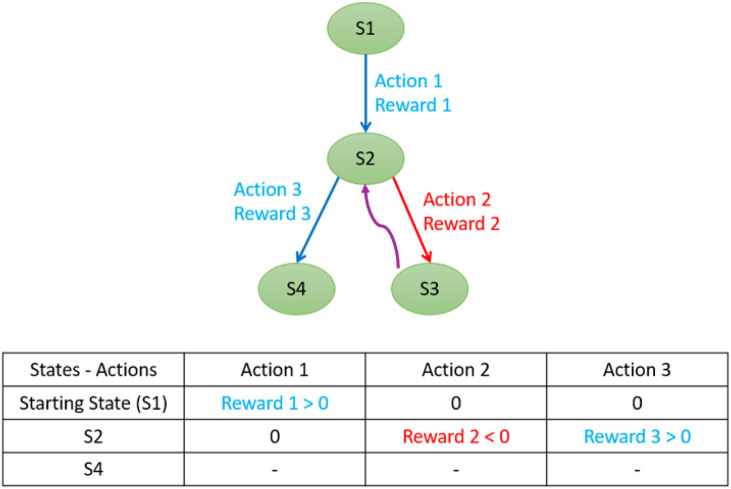
Example of state transition in Q-learning based on action outcomes.

As illustrated in Figure 11, selecting action 2 from state 2 leads to a negative reward because the distance $d_{t}$ increases. Consequently, the algorithm does not transition to state 3 but instead remains in state 2, and the negative reward is recorded for action 2. In the following iteration, the algorithm selects a different action—either by exploiting prior knowledge or through random exploration—and chooses action 3. Since this action results in a smaller $d_{t}$ , it yields a positive reward and transitions the agent to state 4.

If a valid solution—or multiple solutions—exists, the Q-learning algorithm consistently converges toward it as it favors joint adjustment values that minimize the error distance. In cases where no exact solution is possible due to joint failures or constraints, the algorithm outputs a set of joint values and a throwing angle that results in the object being thrown to the position closest to the basket.

- Analysis with sudden constraints.

There are two possible sub-cases in this scenario:

1. Unconstrained action dominance: The reward associated with the unconstrained joint is higher, and the agent consistently selects actions unrelated to constraints. In this case, the algorithm proceeds toward the solution.
2. Constraint-induced deviation (worst case): The agent may choose an action involving a constrained joint either during exploration or due to exploitation, where the constrained joint previously had the highest Q-value. This situation worsens if a sudden constraint (e.g., joint failure) is introduced.

Consider the worst-case scenario where the agent previously selected an action with the highest reward, and the resulting landing point was very close to the basket but not inside it. Suppose a fault occurs in a specific joint, rendering it immobile. Let the following parameters apply:

-
$d_{t}=d_{t+1}=0.02$ m (distance to target remains the same),
-
Q(s,a)=0.5 (previous Q-value),
-
$Q\left(s^{\prime },a\right)=0$ (assuming next state’s value is 0 for simplicity),
- learning rate α=0.5 , and
- discount factor γ=0.5 .

The immediate reward, without any additional reward or penalty, is calculated as follows:

$$R=1-0.5\left(d_{t+1}-d_{t}\right)=1-0.5\left(0.02-0.02\right)=1.$$

The Q-value is updated as follows:

$$Q\left(s,a\right)=0.5+0.5\left(1+0.5\cdot 0-0.5\right)=0.75.$$

The increase in Q(s,a) incorrectly indicates that choosing the same action brings the throwing point closer to the target, which is misleading due to the joint fault.

Reward shaping: To address this issue, additional penalties and rewards are introduced in the reward function:

- If $d_{t+1}\geq d_{t}$ , a large penalty of −20 is applied to discourage moving away from the target.
- If $d_{t+1}<d_{t}$ , a positive bonus of +0.3 is awarded to encourage a reduction in distance.

With the penalty applied, the new immediate reward becomes

$$R=1-0.5\left(0.02-0.02\right)-20=-19.$$

The updated Q-value is

$$Q\left(s,a\right)=0.5+0.5\left(-19+0.5\cdot 0-0.5\right)=-9.25.$$

Compared to the unmodified case, this significantly lower Q-value ensures that the agent avoids selecting the same faulty action in future iterations. Similarly, the positive additional reward helps reinforce beneficial behavior when the distance decreases.

These reward modifications must be carefully tuned to ensure a balance between exploration and exploitation during training.

#### 3.1.3 Q-learning for throwing tasks

The performance of the Q-learning algorithm is evaluated for throwing tasks using a two-link planar arm (Figure 4) and the Franka Emika robot (Figure 5). The experiments are conducted under different scenarios, both with and without boundary constraints.

- Experiment #3: The target basket is placed at varying positions to evaluate the algorithm’s learning capability. A two-link planar manipulator with rotational joints is used, with its DH parameters given in Table 4.

**FIGURE 12:**
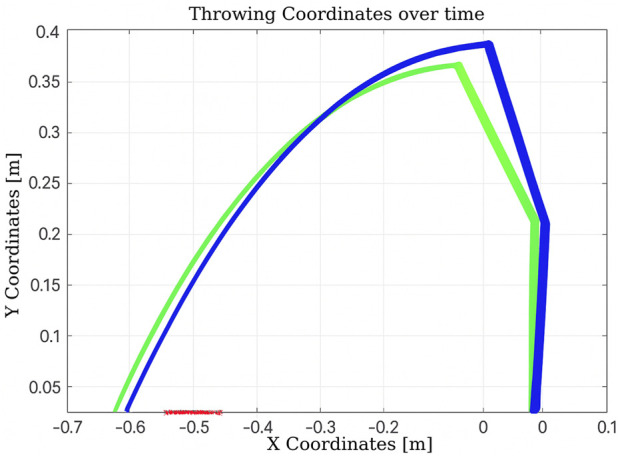
Learning process using Q-learning-based throwing using the two-link planar robot. The figure shows how the robot refines its throwing strategy: the green line denotes the initial attempt, and the blue line reflects improvement in the subsequent throw.

As shown in Figure 12, after the initial throw, the algorithm quickly adapts the manipulator’s joint configuration to bring the ball closer to the basket, using information obtained from the previous throw. The number of iterations is 2, and the elapsed time is 0.010830 s.

**FIGURE 13:**
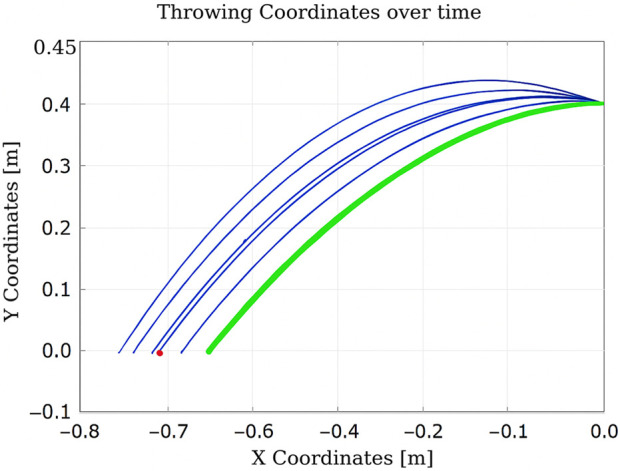
Learning process using Q-learning-based throwing using the two-link planar robot. The figure illustrates the evolution of the learned policy across six training steps, highlighting how the agent progressively improves its decision-making.

In the scenario illustrated in Figure 13, the algorithm executes six actions as follows:

1. Decrease $\theta_{1}$ , negative reward, “randomly.”
2. Increase $\theta_{1}$ , positive reward, “exploit.”
3. Increase $\theta_{1}$ , positive reward, “randomly.”
4. Increase $\theta_{1}$ and $\theta_{2}$ , positive reward, “randomly.”
5. Increase $\theta_{1}$ , positive reward, “exploit.”
6. Increase $\theta_{1}$ , positive reward, “exploit.”

The total elapsed time is 0.143417 s, and the final distance is 0.92 cm.

- Experiment #4: The basket is placed at position (0,0), close to the robot’s base, to evaluate Q-learning behavior under more constrained spatial configurations.

**FIGURE 14:**
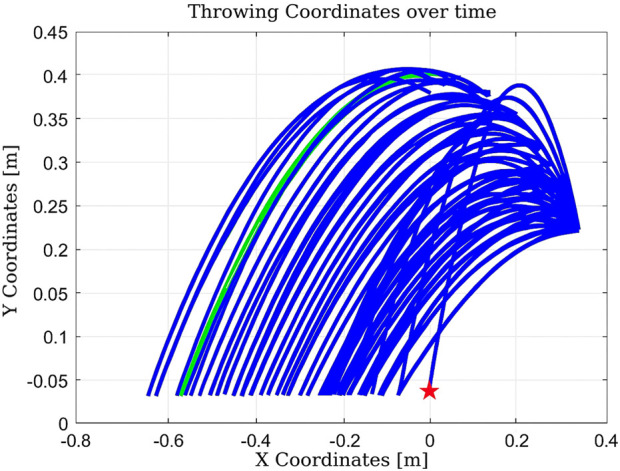
Learning process using Q-learning as the agent progressively improves its actions to reach the target position at (0,0).

As shown in Figure 14, the algorithm finds a valid solution by adjusting the throwing angle. However, it requires a large number of iterations (1183), resulting in a total execution time of 2.248 s (Homsi et al., 2023). This is primarily due to the limited number of states managed by the algorithm.

**FIGURE 15:**
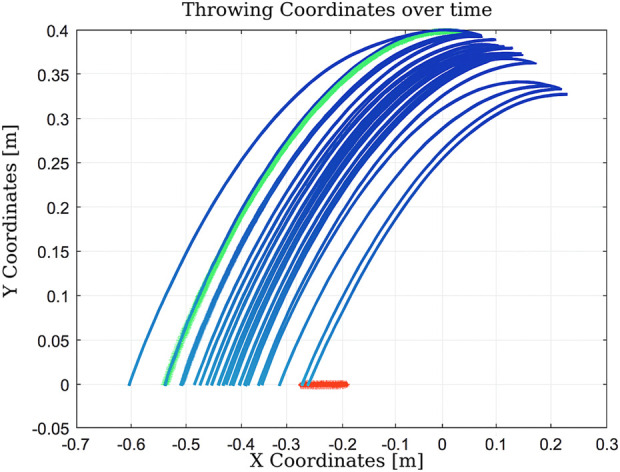
Learning process using Q-learning shows how the agent learns to perform the throwing motion despite being constrained to a limited number of states.

**FIGURE 16:**
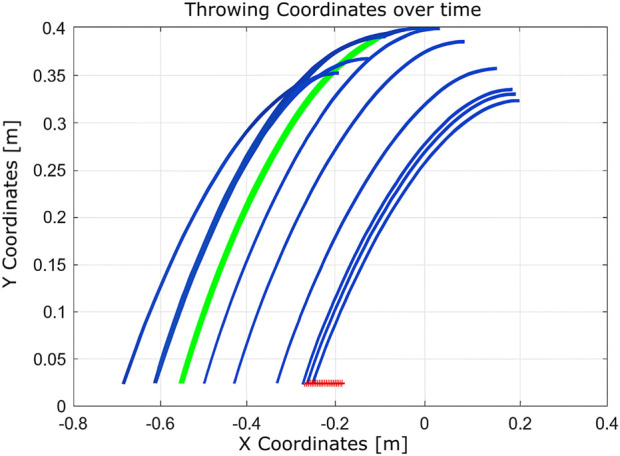
Learning process using Q-learning illustrates improved throwing behavior enabled by incorporating a larger number of states.

**FIGURE 17:**
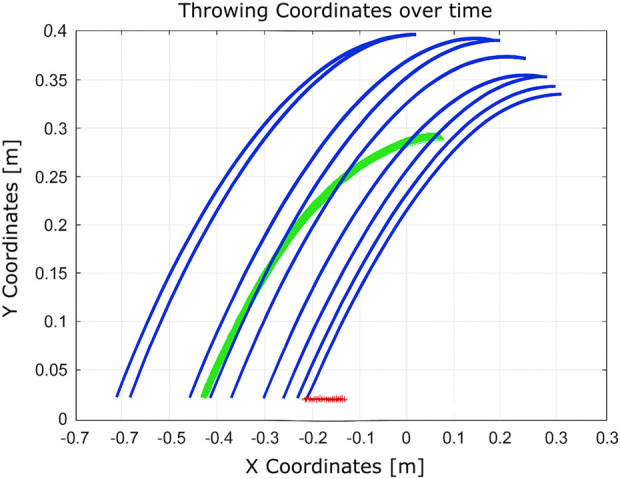
Learning process using Q-learning with more states considered and the fully constrained second joint.

- Experiment #5: This experiment explores performance in three scenarios: (i) a limited number of states (Figure 15), (ii) an expanded state set (Figure 16), and (iii) expanded states with a hardware failure in joint 2 (Figure 17).

These setups assess the algorithm’s adaptability to changes in the state space and the reliability of the hardware. With a limited state space, the algorithm converges in 51 iterations. When the number of states is increased, convergence improves significantly to just nine iterations. However, in the presence of a hardware fault (immobile second joint), convergence requires 12 iterations. This comparison illustrates that expanding the state space enhances learning efficiency, while hardware failures introduce additional complexity that delays convergence.

**FIGURE 18:**
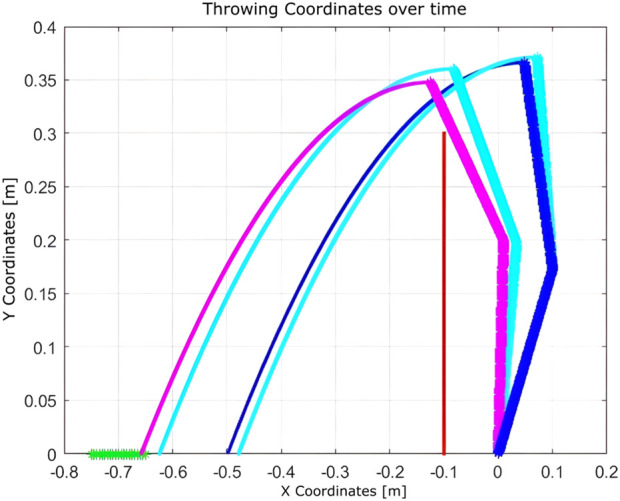
Learning process using Q-learning when a wall presents a nearby boundary.

**FIGURE 19:**
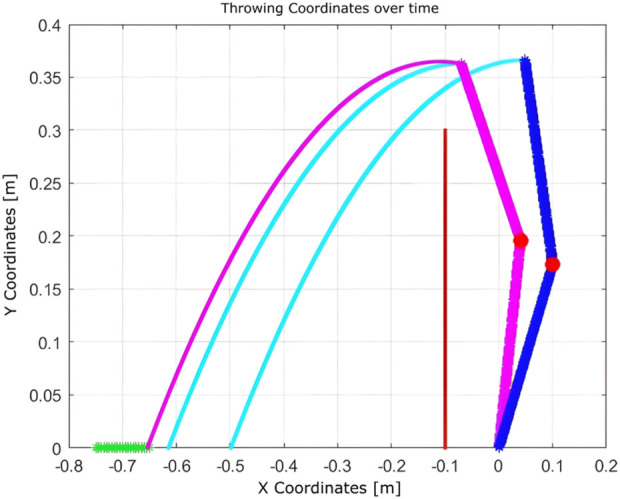
Learning process using Q-learning when there is a wall as a nearby boundary and one joint is completely fixed (nine iterations).

**FIGURE 20:**
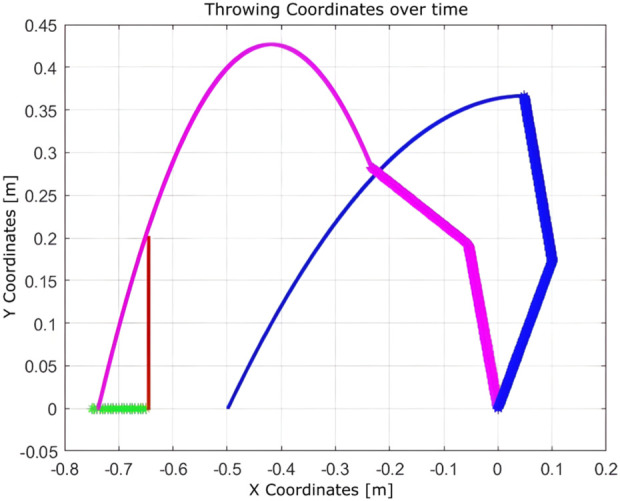
Learning process using Q-learning when a wall presents a boundary that is near the target basket (10 iterations).

- Experiment 6: The algorithm was evaluated under physical constraints, such as the presence of a wall or a nearby person. In certain test scenarios, the algorithm operated without explicitly detecting these constraints, relying solely on the distance between the landing point and the basket, as shown in Figures 18–20.

The algorithm demonstrated notable robustness under the following challenging conditions.

- With a wall near the basket, a successful throw was achieved within 10 iterations.
- In the presence of a hardware failure (immobile second joint) and a nearby wall, the robot successfully adapted, requiring nine iterations.
- When only a wall was present near the robot, the algorithm needed just seven iterations to reach the goal.

These outcomes highlight the adaptability and efficiency of the Q-learning algorithm in environments constrained by physical obstacles and hardware limitations.

- Experiment 7: To further test the adaptability of Q-learning, experiments were conducted using the Franka Emika robot to optimize object throwing into a basket. The robot learned through iterative trials, adjusting its joint parameters to minimize the distance between the landing point and the basket.

**FIGURE 21:**
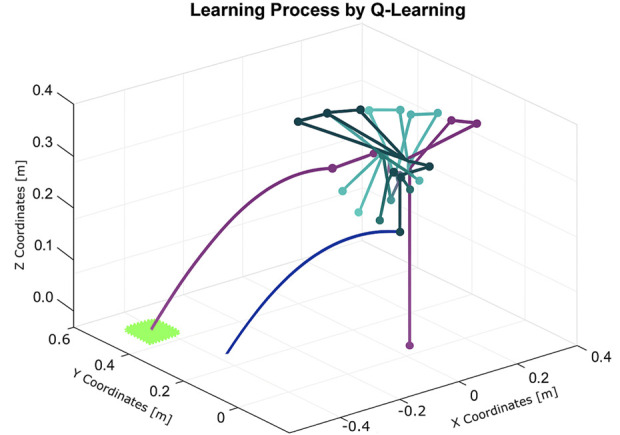
Learning process using Q-learning for the throwing task using the Franka Emika Panda robot (five iterations).

**FIGURE 22:**
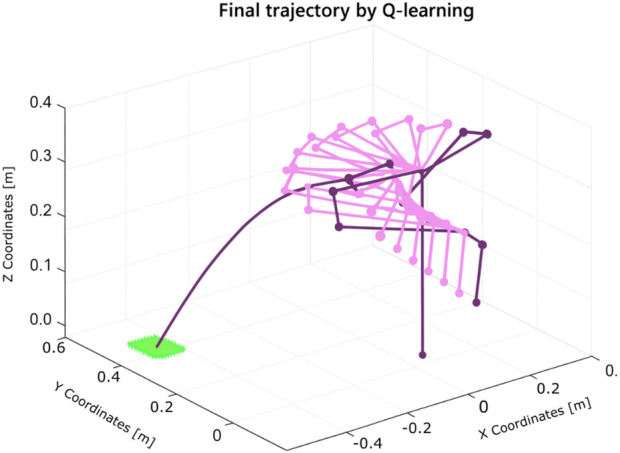
Trajectory of the solution for the throwing task found by Q-learning using the Franka Emika Panda robot.

The learning process, illustrated in Figure 21, demonstrates the robot’s improved performance over time. The resulting trajectory of a successful throw is shown in Figure 22. Remarkably, the algorithm required only five iterations to discover an effective solution using the Franka Emika platform.

Although Q-learning exhibits clear benefits in terms of robustness and rapid convergence, it is important to recognize its limitations in scenarios involving high-dimensional or continuous action spaces. In such cases, deep reinforcement learning methods such as DQNs are typically employed to extend Q-learning’s capabilities (Sutton and Barto, 2018; Watkins, 1989).

### 3.2 Throwing a ball using deep RL

#### 3.2.1 Compatibility with deep learning

Integration with deep neural networks through techniques such as DQNs expands the capabilities of Q-learning (Mnih et al., 2013; Mnih et al., 2015). This combination, often referred to as deep RL, enables Q-learning to handle high-dimensional input spaces, including images, thereby broadening its applicability across diverse domains (Sutton and Barto, 2018; Watkins, 1989).

DQN enhances conventional Q-learning by effectively managing complex and high-dimensional state spaces, which are difficult to represent using tabular methods (Mnih et al., 2013; Mnih et al., 2015). It is a powerful AI technique capable of learning hierarchical features directly from raw input data, eliminating the need for manual feature engineering (Sutton and Barto, 2018; Watkins, 1989).

Despite these advantages, DQNs present several challenges. As an extension of Q-learning, DQN approximates the Q-function using a deep neural network instead of a Q-table. The network takes the environment’s state as input and outputs expected rewards for each possible action. It is trained by minimizing a loss function defined as the difference between predicted and target Q-values. However, DQN involves high computational complexity, requires careful hyperparameter tuning, and may experience instability during training. Additionally, its effectiveness depends on the specific problem characteristics (Sutton and Barto, 2018; Watkins, 1989).

#### 3.2.2 Comparison between DQN and other RL algorithms

**FIGURE 23:**
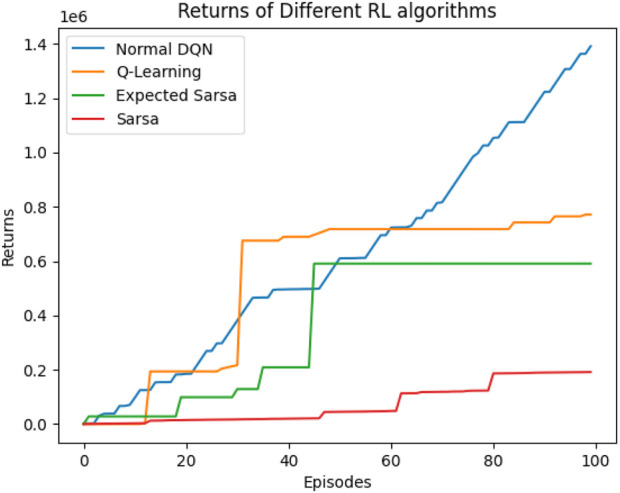
Comparison between DQN, Q-learning, expected SARSA, and SARSA in terms of TAR.

The same experiments were conducted using the standard DQN, and a comparison was made between SARSA, expected SARSA, and Q-learning for the task of throwing a ball into a basket using a two-arm robot manipulator. Upon comparison, DQN demonstrated superior performance over the other algorithms in terms of total average return, as shown in Figure 23.

#### 3.2.3 Comparison between different types of DQNs

**FIGURE 24:**
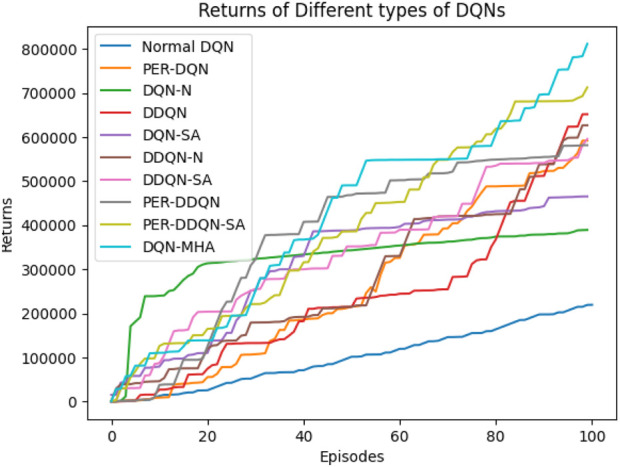
Comparison between different types of DQN in terms of total average return for 100 episodes.

When comparing different types of DQNs for a robot manipulator tasked with throwing balls, several key factors are considered. All types of DQNs succeed in the throwing task; however, DQNs with multi-head attention (DQN-MHA) outperform others in terms of total average return, as shown in Figure 24.

The performance comparison between different DQNs is based on key performance indicators (KPIs) related to policy performance, learning speed, and learning accuracy.

1. Total average return (TAR): this parameter provides an overall measure of the agent’s performance by calculating the mean reward across multiple episodes.
2. Standard deviation (SD): this parameter measures the variability in the reward curve, indicating the consistency of the agent’s performance:
  - Lower SD values indicate more consistent rewards, implying stable agent performance.
  - Higher SD values suggest greater variability in rewards, generally indicating instability or inconsistency in performance.
3. Learning speed: this parameter indicates how quickly the agent improves its performance, how rapidly it converges to an optimal or near-optimal policy, and how effectively it balances exploration and exploitation.
4. Trend slope (TS) of the reward: this parameter reflects the general direction of the agent’s performance over time:
  - Positive TS values indicate increasing rewards, suggesting that the agent is learning and improving, which is desirable.
  - Negative TS values indicate decreasing rewards, implying deterioration in performance, which is generally undesirable.
  - Zero or near-zero TS indicates stable rewards over time, which may indicate a learning plateau.
5. Mean loss: this parameter measures average error; lower values indicate better model performance accuracy.

Based on these KPIs, performance improves as the TAR increases. As shown in Figure 24, DQN-MHA achieves the highest rewards (TAR = 415,505 ), while the standard DQN has the lowest performance (TAR = 100,147 ). Regarding SD and TS, algorithms with lower SD and higher TS are considered superior. As presented in Table 6, DQN-MHA and PER-DDQN-SA outperform other variants in terms of TS. Conversely, standard DQN and DQN-N exhibit the best performance concerning SD. Other DQN types demonstrate intermediate results across KPIs, neither excelling nor significantly underperforming. While they do not lead in all metrics, they maintain competitive performance compared to other methods.

**TABLE 6:** Comparison between different DQNs. The first column lists the considered DQNs. The second column reports the standard deviation of the return, the third column shows the trend slope of the return during training, and the final column indicates the learning speed through the number of episodes required to reach 75%, 90%, and 95% of the final return.

DQN type	SD $\left[10^{5}\right]$	TS $\left[10^{4}\right]$	75%	90%	95%
DQN-MHA	2.25	0.766	86	95	98
DQN-SA	1.435	0.4625	84	94	97
DQN-N	0.793	0.288	79	92	96
PER-DDQN-SA	2.126	0.733	86	95	98
DDQN-SA	1.6	0.546	85	95	98
DDQN-N	1.745	0.595	88	96	98
DDQN	1.727	0.572	91	97	99
PER-DDQN	1.973	0.636	84	94	97
PER-DQN	1.883	0.647	89	96	98
Standard-DQN	0.664	0.227	89	97	99

Another crucial metric is learning speed, which indicates how rapidly an agent improves its performance during training. It is typically measured by the rate at which cumulative reward or average reward per episode increases over time. Common measurements include the number of episodes needed to reach 75% , 90% , and 95% of the total reward. Learning speed generally depends on factors such as the neural network architecture, the quality of the training data, and the effectiveness of the exploration–exploitation strategy (Li, 2023; Lapan, 2018). The results from the previous experiment are summarized in Table 6. Based on the mean loss KPI, the results demonstrate a significant reduction in mean loss from the standard DQN (242.2) to the enhanced variants. DDQN-based models demonstrate significantly lower losses, with DDQN-N achieving the lowest among standard variants (0.26). PER-enhanced methods further improve performance by several orders of magnitude, with PER-DQN reaching as low as $7.367\times 10^{-12}$ , indicating more stability and learning efficiency, as summarized in Table 7.

**TABLE 7:** Mean loss values for different DQNs. The mean is computed as $\mu =\frac{1}{n}\sum_{i=1}^{n}x_{i}$ .

Algorithm	Mean loss (μ)
DQN	242.2
DQN-MHA	211.05
DQN-N	142
DQN-SA	104.36
DDQN	6.6
DDQN-SA	0.34
DDQN-N	0.26
PER-DQN	$7.367\times 10^{-12}$
PER-DDQN	$7.5458230\times 10^{-8}$
PER-DDQN-SA	$1.22712\times 10^{-10}$

Based on these results, DQN-N emerges as the best-performing algorithm. By injecting noise into network parameters, DQN-N enhances exploration by introducing stochasticity in action selection. This enables the agent to explore a broader range of actions and states more effectively, accelerating the discovery of optimal policies. Additionally, DQN-N reduces dependence on manually tuned parameters such as ϵ , leading to more stable and consistent learning. By balancing exploration and exploitation while minimizing hyperparameter tuning, DQN-N achieves superior learning speed, making it a powerful alternative among DQN variants (Li, 2023; Lapan, 2018).

#### 3.2.4 Throwing an object to an unknown position of the basket using DQNs with and without an external constraint

**FIGURE 25:**
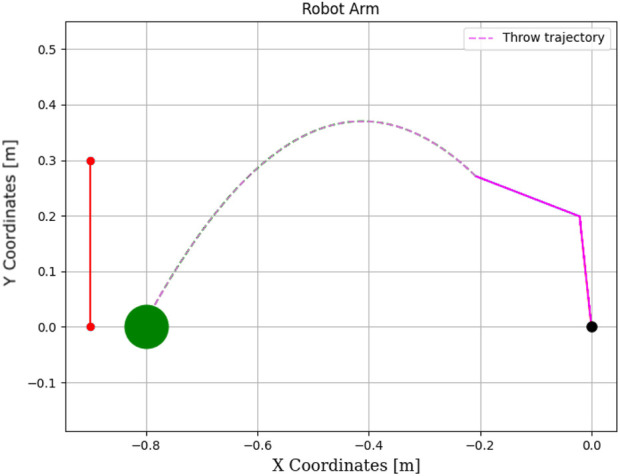
Throwing task using the DQN without a wall.

**FIGURE 26:**
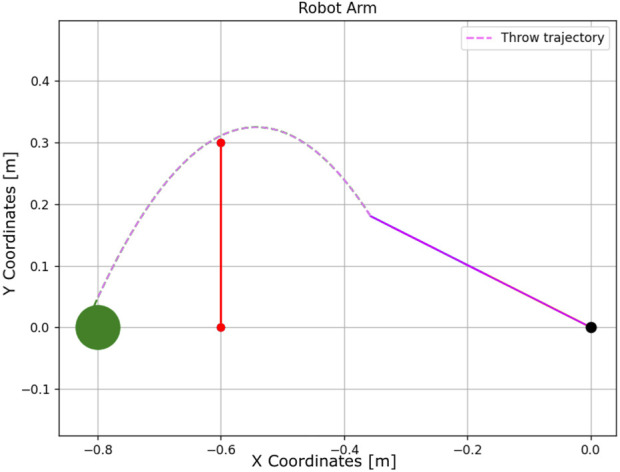
Throwing task using the DQN with a wall.

To evaluate the robustness of various DQN algorithms in uncertain conditions, two experiments were conducted. In both cases, DQNs were trained to throw a ball into a basket located 0.6 m away from the robot manipulator. Their performance was then tested without retraining when the basket was moved to a new location, 0.8 m away from the robot. The first experiment was performed without any obstacles, while the second included a wall obstructing the throw, as illustrated in Figures 25, 26. Each algorithm was tested over 100 trials using the same robot manipulator. The training settings (hyperparameters, the activation function, and optimizer) were consistent across all DQNs and are listed below:

- Learning rate = 0.001
- Network layers = 12
- Episodes = 100
- Neurons for each layer = 512
- Epoch = 500
- Activation function = ReLU
- Optimizer = Adam

The performance results are summarized in Table 8, showing the number of successful throws into the relocated basket with and without the wall for each DQN variant.

**TABLE 8:** Comparison between different DQNs (task performance). The first column lists the considered DQNs. The second column shows the number of attempts required to successfully throw the ball to a new target location without a wall. The third column presents the number of attempts needed when a wall is introduced as an obstacle.

DQN type	No boundaries	With boundaries
DQN-MHA	18	35
DQN-SA	23	39
DQN-N	25	52
DDQN	25	53
PER-DQN	24	45
DDQN-N	26	55
PER-DDQN	24	45
PER-DDQN-SA	21	39
DDQN-SA	23	49
Standard-DQN	31	68

The results indicate that DQN-MHA demonstrated the best overall performance in both scenarios. This suggests that the multi-head attention mechanism significantly enhances the network’s ability to focus on important environmental features. This attention-driven adaptability enables DQN-MHA to outperform other variants by achieving higher precision in altered environments, particularly when obstacles are introduced.

## 4 Discussion

### 4.1 Results and discussion

The experiments were repeated using the best-performing configuration for each algorithm, with hyperparameters tuned individually. To ensure optimal performance of each algorithm, we employed Optuna, a modern open-source framework for automated hyperparameter optimization that enables efficient exploration of the hyperparameter space and is recognized as one of the most effective tools for this purpose. Specifically, we utilized Bayesian optimization via the tree-structured Parzen estimator (TPE), a variant of sequential model-based optimization (SMBO), to guide the search process. To enhance robustness and mitigate overfitting, cross-validation was integrated into the optimization pipeline. The final configurations, summarized in Table 10, correspond to the best-performing hyperparameter sets identified by Optuna for each algorithm, as shown in Table 9.

**TABLE 9:** Hyperparameter settings for DQN variants in reinforcement learning experiments. Optimized hyperparameters are used for each algorithm. The learning rate α is shown in the column labeled $\alpha \;\left[10^{-3}\right]$ . The number of hidden layers is specified in the column $n_{l}$ , while $n_{\mathrm{neuron}}$ denotes the number of neurons per layer. The activation function and optimizer are the same across all DQNs. The same number of neurons was used across all layers within a given model.

DQN type	α ( $10^{-}3$ )	$n_{l}$	$n_{\mathrm{neuron}}$
DQN-MHA	10	256	2024
DQN-SA	10	128	1024
DQN-N	0.3	8	256
DDQN	0.1	8	512
PER-DQN	0.5	10	512
DDQN-N	0.5	8	256
PER-DDQN	0.5	8	256
PER-DDQN-SA	1	12	512
DDQN-SA	5	12	512
Standard-DQN	0.1	12	512

All algorithms were optimized with the same procedure (Optuna with Bayesian TPE, equal trial budgets, identical metrics, and cross-validation). Search spaces were adapted to each algorithm since DRL methods differ in terms of stability, sensitivity, and capacity requirements; for instance, attention-based variants often require deeper networks, while vanilla DQN is more susceptible to high learning rates as they cause Q-values to fluctuate excessively (each update potentially overwriting previous estimates), leading to divergence or oscillations in the Q-function. Imposing identical ranges would have biased results by forcing some methods into suboptimal regions rather than allowing them to explore the whole search space. Fairness was ensured by giving every algorithm equal opportunity to reach its best configuration within an appropriate search space, so the observed differences in hyperparameters in Table 9 reflect intrinsic inductive biases rather than unequal treatment, as shown in Table 9.

**FIGURE 27:**
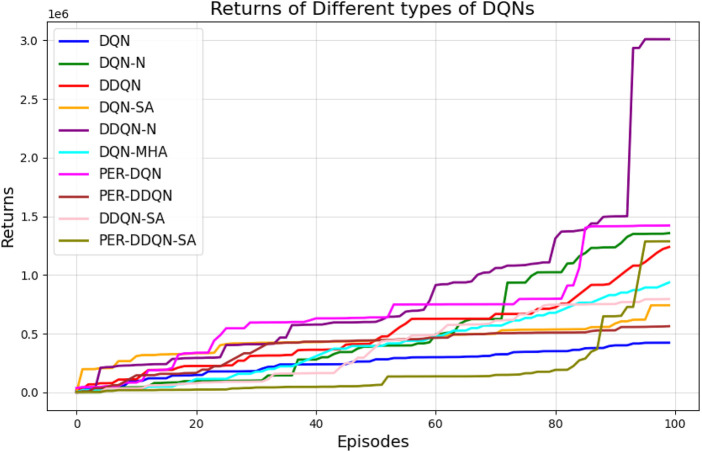
Comparison between different types of DQNs in terms of total average return for 100 episodes after tuning.

Figure 27 illustrates the performance of various DQN variants in terms of TAR, following individual tuning. The TAR metric evaluates the cumulative rewards collected across episodes during training, offering a quantitative measure of policy effectiveness. In robotic manipulation tasks, a higher TAR reflects the policy’s ability to consistently execute precise and repeatable throwing trajectories.

Among the evaluated algorithms, *DDQN-N* achieved the highest TAR value of 3,010,079 , surpassing even the performance of *PER-DDQN-SA*. The superior performance of *DDQN-N* can be attributed to the following factors:

- Stochastic policy exploration: The use of noisy weights in noisy DQN allows for adaptive exploration during training, enhancing the agent’s ability to navigate the complex and high-dimensional reward landscape of the throwing task.
- Robust value estimation: Double Q-learning mitigates overestimation bias by decoupling action selection and evaluation, resulting in more reliable Q-value estimates and fewer suboptimal decisions in environments with high variance.

The poor performance of the standard *DQN* (424,286) can be attributed to the following factors:

- Over-reliance on greedy policies: Standard DQN primarily follows greedy action selection, which limits exploration and prevents the agent from discovering better strategies.
- Inadequate handling of reward sparsity: The throwing task often involves delayed and sparse rewards, requiring effective temporal credit assignment. Standard DQN struggles to propagate reward signals across long episodes, resulting in suboptimal learning.

Attention-based variants, such as *DQN-MHA* (937,760) and *DQN-SA* (743,742) , show moderate improvements in TAR. These architectures benefit from hierarchical representation learning, where attention mechanisms excel at identifying critical dependencies in throwing dynamics—essential for accurate trajectory prediction. However, in the absence of other key enhancements—such as double Q-learning to reduce overestimation or noisy networks to encourage better exploration—their performance remains limited, with slower convergence and reduced robustness compared to more advanced variants.

**FIGURE 28:**
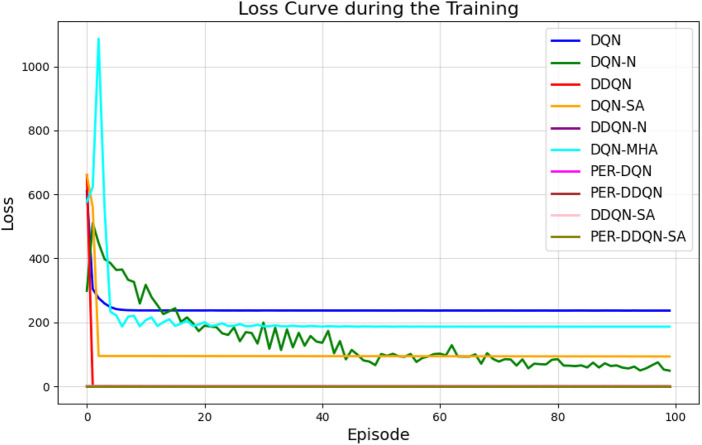
Loss curves for all DQN architectures across training episodes, illustrating convergence behavior.

**FIGURE 29:**
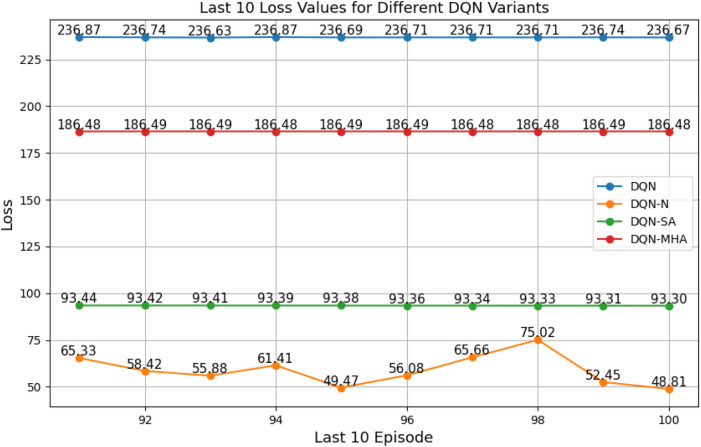
Last 10 loss values for DQN, DQN-N, DQN-MHA, and DQN-SA architectures.

**FIGURE 30:**
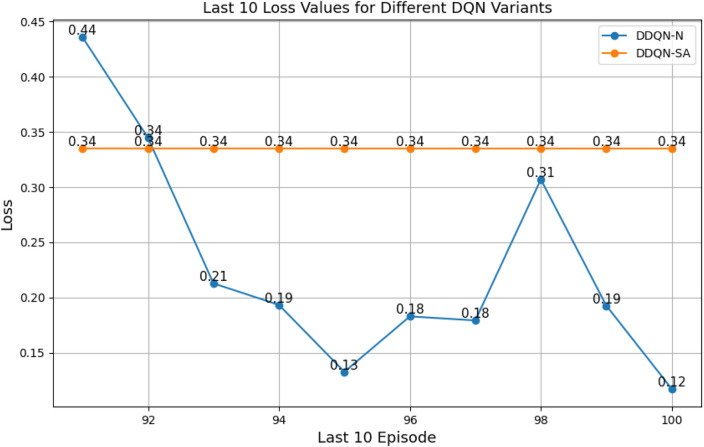
Last 10 loss values for DDQN-SA and DDQN-N architectures.

**FIGURE 31:**
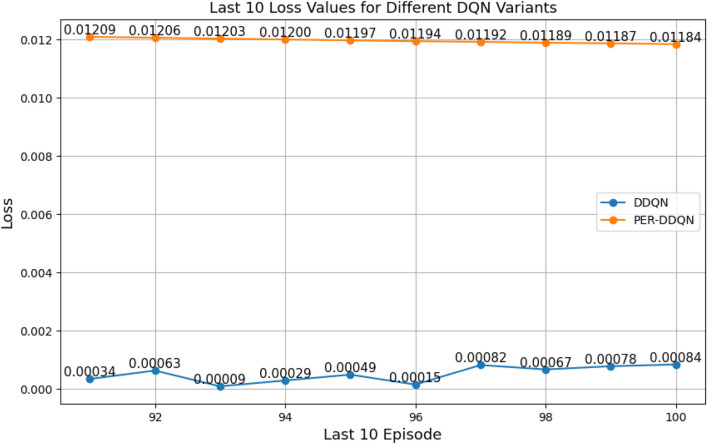
Last 10 loss values for PER-DDQN and DDQN architectures.

**FIGURE 32:**
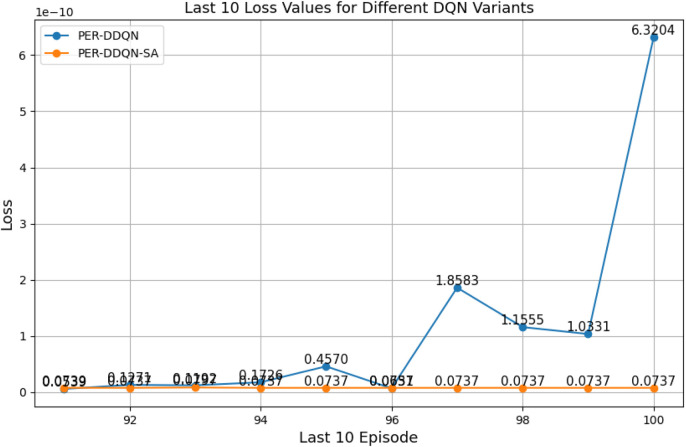
Last 10 loss values for PER-DQN and PER-DDQN-SA architectures.

The loss curve, or training curve, illustrates the temporal difference (TD) error during the learning process. This metric captures the discrepancy between the estimated value function and the actual return received from the environment. As shown in Figures 28–32, the TD error provides insights into the convergence behavior and stability of different DQN variants during training.

Among all evaluated algorithms, the standard *DQN* exhibited the poorest performance, with the highest loss value (236.67), indicating instability in the learning process. This poor performance is attributed to overestimation bias, a known limitation of standard DQN due to the absence of enhancements such as target networks with double estimation or structured noise. In contrast, advanced variants—such as *PER-DQN*, *PER-DDQN*, and the self-attention-based *PER-DDQN-SA*—achieved significantly lower loss values, with *PER-DDQN-SA* reaching the lowest at $6.32\times 10^{-10}$ . These results can be explained by the improved training efficiency introduced by prioritized experience replay. Unlike standard DQN, which samples past transitions uniformly, PER prioritizes training on transitions with high temporal-difference error. This prioritization emphasizes informative yet rare events—such as successful or failed throws—that are critical in tasks such as robotic ball-throwing.

By sampling more frequently from these high-value experiences, PER guides the learning process toward more effective policy updates, especially in environments characterized by sparse rewards and high-dimensional state–action spaces. Furthermore, the *DDQN* architecture addresses the overestimation bias by decoupling action selection from value estimation using two separate networks. When combined with structured exploration strategies such as noisy networks, *DDQN-N* demonstrated notable stability (0.1171), suggesting that guided stochastic exploration accelerates convergence in complex robotic control tasks.

As shown in Table 10, *PER-DDQN* and *DDQN-N* required the fewest number of trials to successfully throw the ball to a new location without hitting a wall. This highlights the algorithms’ superior ability to generalize the task dynamics. The use of prioritized experience replay enables more efficient learning from informative transitions, while the incorporation of double Q-learning mitigates overestimation bias during value updates. Moreover, the inclusion of noisy weights in the DQN-N variants further improves the exploration–exploitation trade-off by enabling more structured and adaptive exploration, outperforming algorithms that rely on fixed or simplistic exploration strategies.

**TABLE 10:** Comparison between different DQNs after hyperparameter optimization. The first column lists the considered DQNs. The second column shows the number of attempts required to reach a new target without a wall, while the third column shows the number of attempts required with a wall in place.

DQN type	No boundaries	With boundaries
DQN-MHA	16	30
DQN-SA	21	35
DQN-N	16	29
DDQN	15	29
PER-DQN	16	35
DDQN-N	12	27
PER-DDQN	12	28
PER-DDQN-SA	20	37
DDQN-SA	20	46
Standard-DQN	29	61

In contrast, standard *DQN* consistently demonstrated poor performance across multiple metrics, including high loss, low TAR, and inefficient policy convergence. These shortcomings are primarily due to its reliance on basic epsilon-greedy exploration, which often results in overfitting and suboptimal Q-value estimation. Notably, DQN variants with attention mechanisms achieved higher trial efficiency compared to their non-attention counterparts. In particular, *DDQN-SA* benefited from the ability to better reason about complex dependencies, such as the relationship between ball trajectories and basket locations, leading to more effective decision-making during task execution.

The combined techniques with standard DQN, such as double networks, prioritized experience replay, noisy networks, and self-attention, show better convergence, higher TAR, and greater trial efficiency. These findings reflect the importance of many structured exploration and reasoning mechanisms in DRL for complex robotic tasks such as throwing.

### 4.2 Robustness evaluation

To evaluate robustness to sensor noise and joint constraints, we choose two architectures: DQN as a baseline architecture and DDQN-N as an architecture with superior performance.

#### 4.2.1 Testing sensitivity to sensor noise

To evaluate the robustness of the trained DQN and DQN-N policies under sensor noise, we introduced zero-mean Gaussian noise to the input observations during testing—specifically to the joint positions and the computed throwing angle. The noise was applied with standard deviations of 0.01, 0.05, and 0.1, as suggested.

**FIGURE 33:**
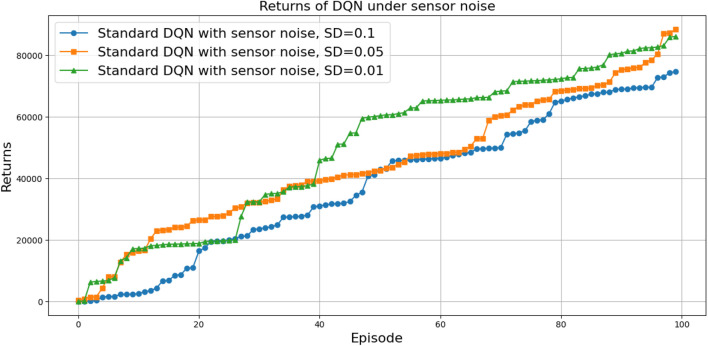
Comparison of the total average return for the DQN architecture under different levels of sensor noise.

**FIGURE 34:**
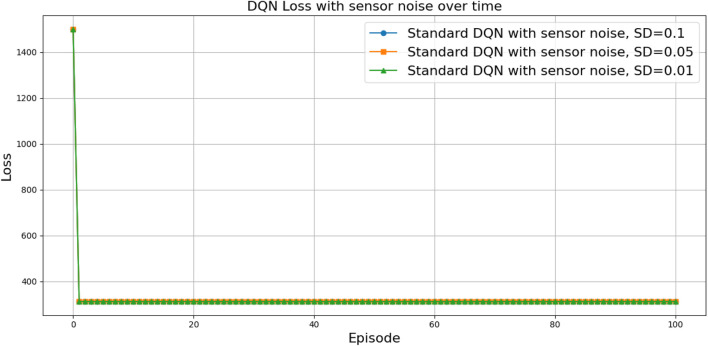
Loss curves for the DQN under different levels of sensor noise.

**FIGURE 35:**
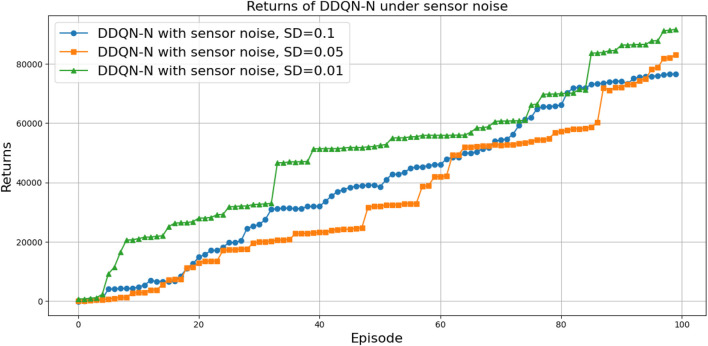
Comparison between DDQN-N for different levels of sensor noise in terms of the total average return.

**FIGURE 36:**
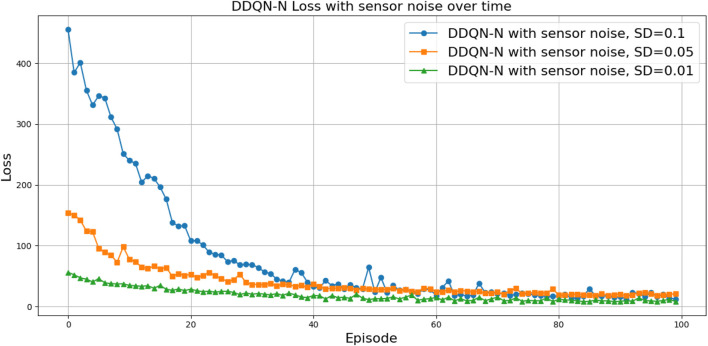
Loss curves for DDQN-N under sensor noise.

The performance under these noise levels is illustrated in Figures 33, 34 for DQN and Figures 35, 36 for DDQN-N, which show that all three policies maintain similar TAR and loss curve behavior across noise levels. However, differences become apparent when examining the number of attempts required to complete the task.

**FIGURE 37:**
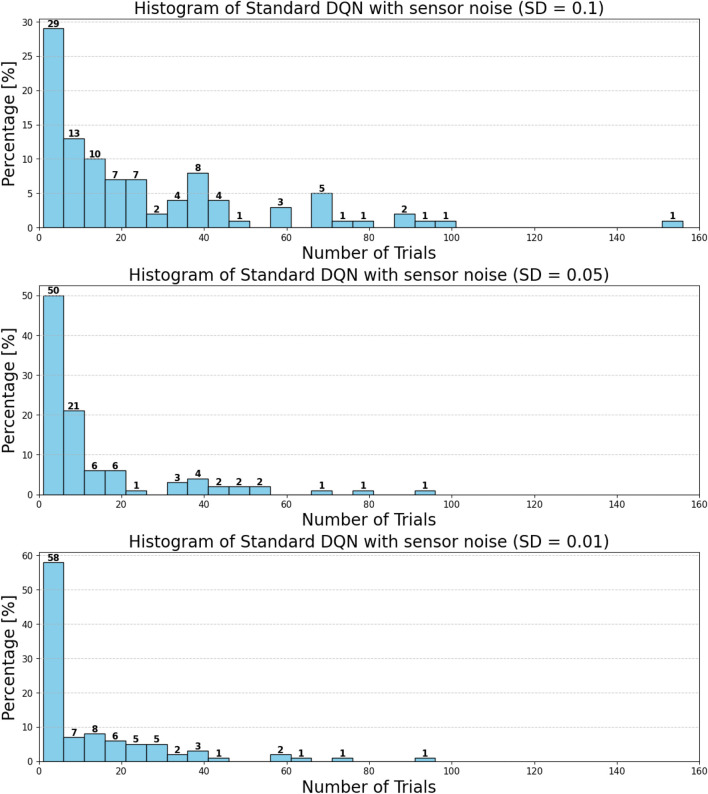
Histogram of attempts needed to successfully throw using DQN under sensor noise. In each episode (out of 100 in total), the robot has different initial positions.

**FIGURE 38:**
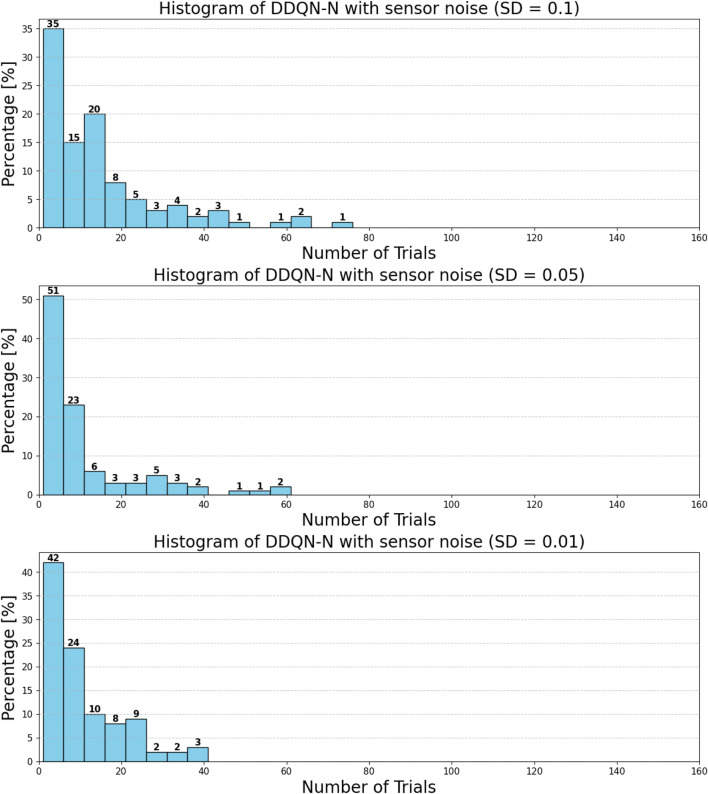
Histogram of attempts needed to throw using the DDQN-N under sensor noise. In each episode (out of 100 in total), the robot has different initial positions.

For this evaluation, we conducted 100 tasks for each DQN architecture. In each task, the planar robot arm started from a different initial position and attempted to throw an object toward a new target. The number of attempts required before a successful throw was recorded and plotted as a histogram, shown in Figures 37, 38 for DQN and DDQN-N, respectively. Table 11 summarizes the results for DQN and DDQN-N. The pairwise comparison results show that both the DQN and DDQN-N algorithms are sensitive to increases in sensor noise, particularly when comparing high noise levels (SD = 0.10) with lower levels (SD = 0.05 and SD = 0.01). For DQN, these differences are statistically significant with medium effect sizes, indicating a clear degradation in performance as noise increases, as shown in Table 12. In contrast, DDQN-N also shows significant differences under the same comparisons, but with smaller effect sizes, indicating greater robustness to noise perturbations. Comparisons between SD = 0.05 and SD = 0.01 are not significant for either algorithm, implying that performance remains stable at lower noise levels. Overall, DQN is more strongly impacted by noise, whereas DDQN-N exhibits greater resilience, though it still exhibits measurable performance degradation under high-noise conditions, as shown in Table 13.

From these results, two key observations can be made:

- Impact of noise level: All architectures performed better with lower noise levels. As sensor noise increased, a higher proportion of throws required more attempts.
- Comparison between DQN and DDQN-N: DDQN-N showed improved resilience to noise. For instance, under the highest noise level (with a standard deviation of 0.1), only 8% of the throws with DDQN-N required more than 40 attempts, compared to 20% for the baseline DQN.

These results indicate that DDQN-N exhibits greater robustness to input noise. We attribute this to the architectural differences: standard DQN learns a deterministic state–action mapping via the Q-function, which makes it more susceptible to perturbations in the input. In contrast, DDQN-N integrates mechanisms that enable it to better generalize over noisy observations.

#### 4.2.2 Testing sensitivity to joint constraints

To evaluate the impact of joint constraints on throwing task performance, we considered a two-link planar robot and imposed the following constraints:

- The angles of both joints are limited to move within the range $\left[0,\frac{\pi }{6}\right]$ .
- The angles of both joints are limited to move within the range $\left[\frac{\pi }{6},\frac{\pi }{3}\right]$ .
- The angles of both joints are limited to move within the range $\left[\frac{\pi }{3},\frac{\pi }{2}\right]$ .

We again employed DQN and DQN-N architectures and compared their performance.

**FIGURE 39:**
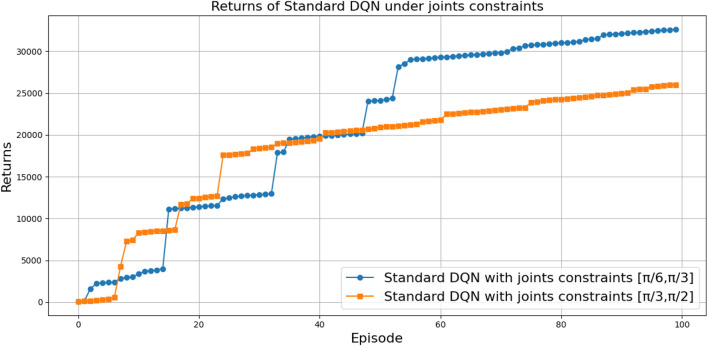
Comparison between DQN for two different joint constraints in terms of the total average return.

**FIGURE 40:**
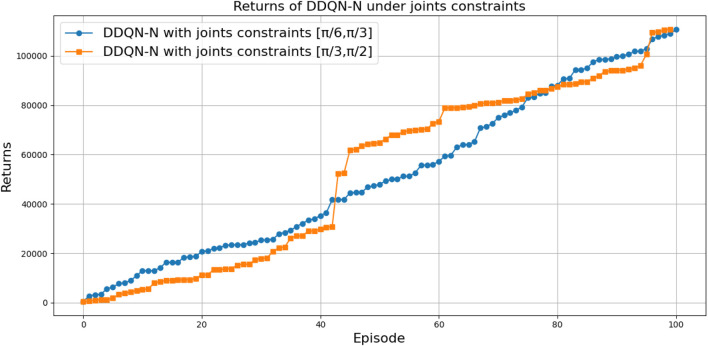
Comparison between DDQN-N for two different ranges of the joint constraints in terms of the total average return.

**FIGURE 41:**
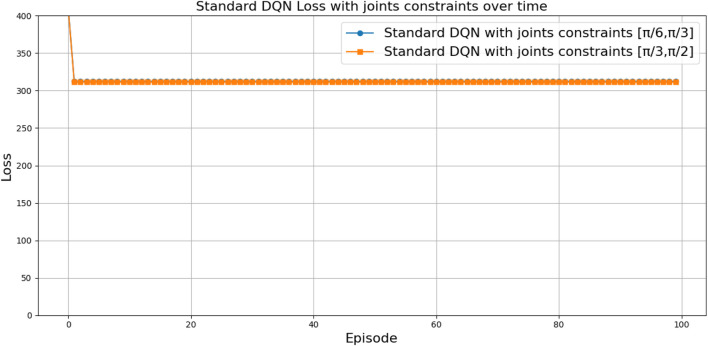
Loss curves for the DQN under joint constraints.

**FIGURE 42:**
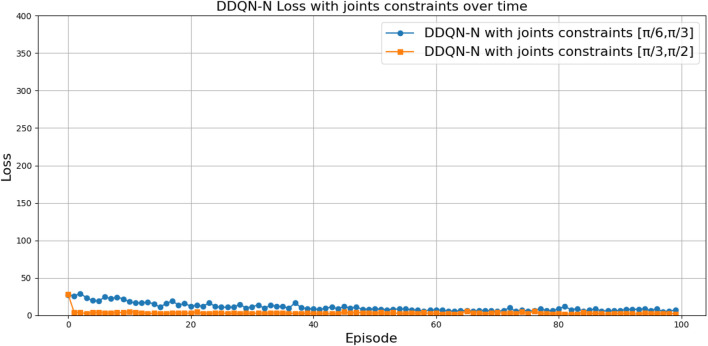
Loss curves for the DDQN-N under joint constraints.

**FIGURE 43:**
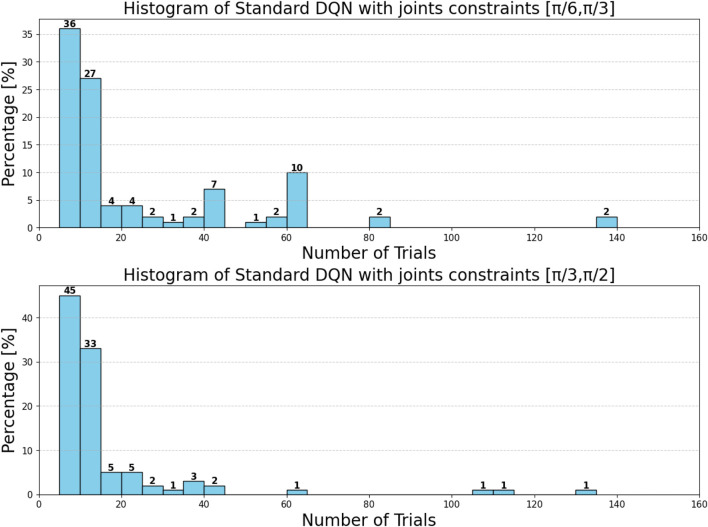
Histogram of iterations needed to throw using the DQN under joint constraint.

**FIGURE 44:**
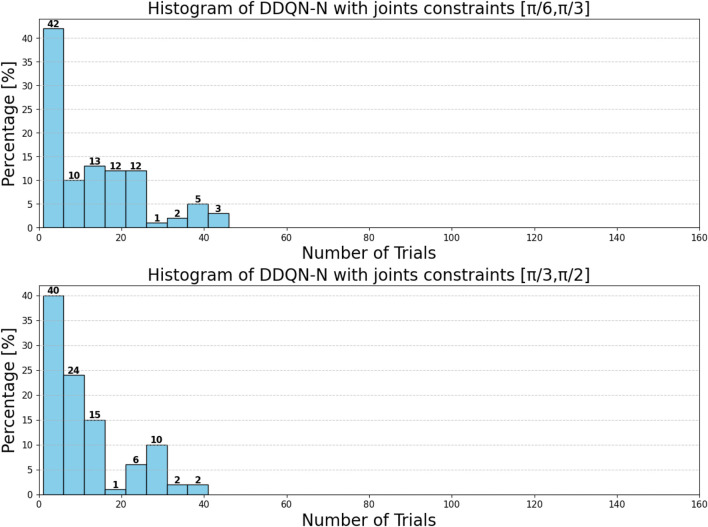
Histogram of iterations needed to throw using the DDQN-N under joint constraint.

We could observe the following:

- The range of the joint constraint highly impacts the robot’s performance. For example, in the case of the first range, neither DQN nor DDQN-N could find the solution and the task has consistently failed. On the other hand, for the other two ranges, both DQN and DDQN-N were able to successfully learn the task.
- For successful ranges, the evolution of the total average return (Figures 39, 40) and the loss curves (Figures 41, 42) were similar across all architectures. However, there was a notable difference in the loss curve value: for DQN, it exhibited high values (above 311), indicating slower convergence and greater variance in Q-value estimates, whereas for DDQN-N, it converged to 0.
- When it comes to the number of attempts needed to successfully throw the ball, both architectures had a relatively high and consistent performance (see Figures 43, 44). However, DDQN-N had no throws where the number of attempts was greater than 50.

The joint range comparison highlights differing sensitivities between the DDQN-N and DQN algorithms. For DDQN-N, the performance difference between the 30°–60° and 60°–90° ranges is not statistically significant, with a very small effect size, indicating stable behavior across these ranges. In contrast, DQN shows a significant difference at the 95% confidence level, with a small-to-medium effect size, suggesting that its performance is more strongly influenced by joint range variations. These results suggest that DDQN-N demonstrates greater robustness to changes in joint range, while DQN is more susceptible to performance shifts under different configurations, as shown in Table 15.

In summary, DDQN-N showed faster reward convergence, lower loss, and overall more reliable performance than DQN, as shown in Table 14.

### 4.3 Analysis of the time window

In this section, we examine how the sequence length influences the performance of the DQN-MHA architecture. In this context, the sequence length refers to the number of consecutive time steps provided as input to the self-attention mechanism for the assigned task. As this parameter directly influences how the model integrates past observations (previous knowledge) when estimating Q-values for the current state, it affects both short-term reactivity and long-term planning. The shorter sequences improve efficiency and reactivity over time but may fail in long-term tasks, and longer sequences enhance strategic foresight but require more computation and data.

To evaluate the impact of the sequence length on the performance of DQN-MHA, different values of the sequence length are used (Ba et al., 2016; Britt, 2020; Hausknecht and Stone, 2015; Mnih et al., 2015) as follows:

- Sequence length = 4: With a very short context window, the DQN captures only the most recent states, enabling fast computation and reduced memory usage due to smaller attention matrices, resulting in fast training. However, it limits the modeling of delayed rewards, often resulting in short-sighted strategies in multi-step planning.
- Sequence length = 8: It has better performance than length 4 as it provides more historical context to DQN. On the other hand, it required greater computational and memory resources than length 4 and may still fail in tasks requiring long-term credit assignment.
- Sequence lengths = 16 and 32: It allows attention to operate over an extended history, enabling the capture of long-term dependencies and delayed rewards. However, it needs greater computational and memory resources for attention operations ( $O\left(n^{2}\right)$ complexity) and carries a higher risk of overfitting in small or simple environments.

**FIGURE 45:**
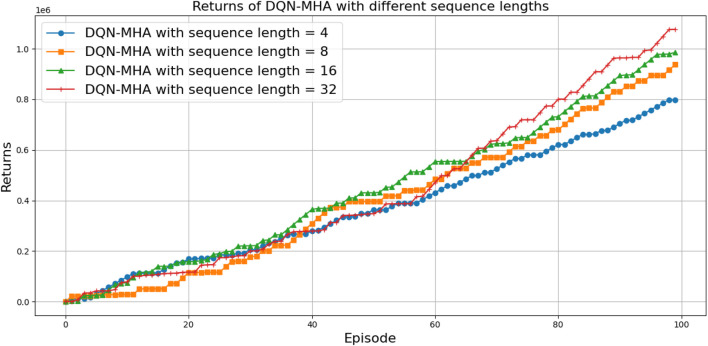
Comparison between DQN-MHA for different sequence lengths in terms of the total average return.

**FIGURE 46:**
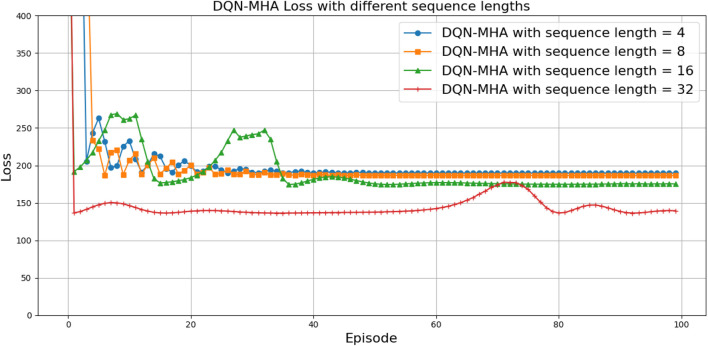
Loss curves for DQN-MHA for different sequence lengths.

**FIGURE 47:**
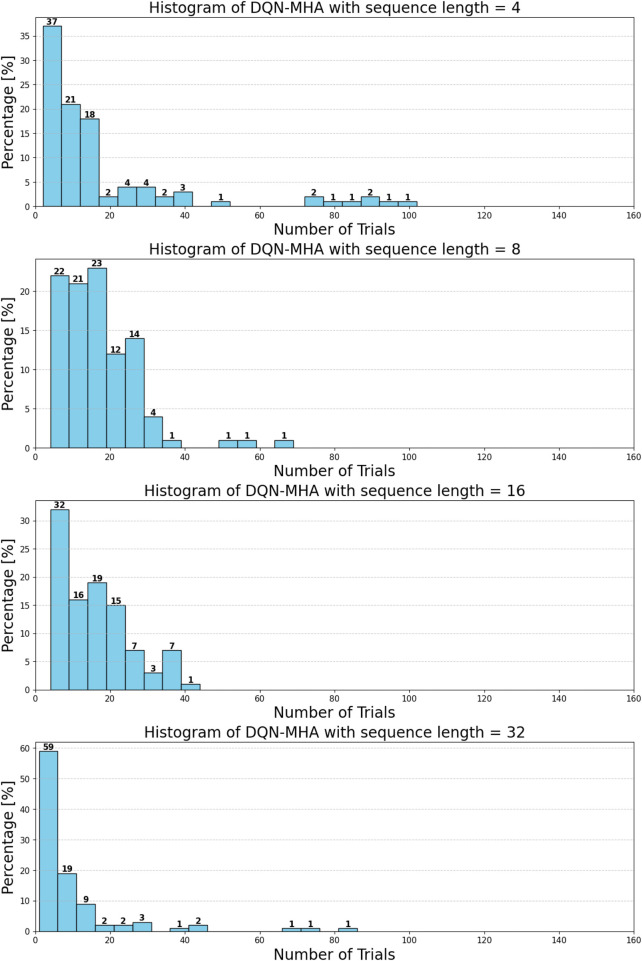
Histogram of iterations needed to throw using the DQN-MHA with different sequence lengths.

Regarding the TAR and loss curves (see Figures 45, 46), all architectures show similar behavior with a slight improvement observed for DQN-MHA using a 32-time-step window, as shown in Table 16. However, the results in Figure 47 show that increasing the sequence length generally improves the efficiency of the DQN-MHA. When the sequence length increases from 4 to 8, the agent’s average decreases from 17.51 to 16.01 and further to 15.08 with a sequence length of 16, showing gradual gains as more contextual information becomes available to the agent. The largest improvement occurred at 32 steps, where the average decreased sharply to 9.25 trials. This suggests that the assigned task benefits from long context, which helps model the delayed rewards for more consistent planning. Overall, increasing the sequence length in DQN-MHA improves performance, particularly when changing from medium to long context windows. However, this gain comes at the expense of higher computational and memory usage. The DQN-MHA results show no significant differences among shorter windows (TW = 4, 8, and 16), with negligible effects. In contrast, TW = 32 consistently outperforms the others, yielding significant improvements with medium effect sizes, as shown in Table 17.

To ensure statistical robustness, all reported results are based on N independent runs with different random seeds. In addition to mean values, we provide 90%, 95%, and 99% confidence intervals (Tables 11–17), including the corresponding half-widths, which quantify the uncertainty associated with each estimate. Reporting confidence intervals across multiple levels offers information equivalent to formal significance testing while avoiding the pitfalls of assuming specific distributional forms, which are often violated in reinforcement learning outcomes. The observed performance differences, therefore, represent statistically meaningful improvements. Furthermore, the magnitude of gains (e.g., DQN-MHA achieving approximately 60% higher mean performance than DQN under comparable conditions) illustrates the practical significance of the results. Our choice of sample size follows established practice in the reinforcement learning literature, balancing computational feasibility with reliable estimation of performance variability.

**TABLE 11:** Performance with 90%, 95%, and 99% confidence intervals of the number of trials across algorithms against sensor noise. h denotes the half-width of the CI.

Algorithm	90%		95%		99%	
	Mean ±h	CI	Mean ±h	CI	Mean ±h	CI
DQN, SD = 0.01	12.22±2.88	(9.34, 15.1)	12.22±3.44	(8.78, 15.66)	12.22±4.56	(7.66, 16.78)
DQN, SD = 0.05	12.52±2.98	(9.54, 15.5)	12.52±3.56	(8.96, 16.08)	12.52±4.71	(7.81, 17.23)
DQN, SD = 0.1	24.76±4.57	(20.19, 29.33)	24.76±5.46	(19.3, 30.22)	24.76±7.23	(17.53, 31.99)
DDQN-N, SD = 0.01	9.99±1.57	(8.42, 11.56)	9.99±1.87	(8.12, 11.86)	9.99±2.48	(7.51, 12.47)
DDQN-N, SD = 0.05	10.22±2.17	(8.05, 12.39)	10.22±2.6	(7.62, 12.82)	10.22±3.44	(6.78, 13.66)
DDQN-N, SD = 0.1	14.55±3.0	(12.04, 17.06)	14.55±3.0	(11.55, 17.55)	14.55±3.97	(10.58, 18.52)

**TABLE 12:** Pairwise comparisons of the DQN algorithm under different sensor noise levels. Mean differences (Δ) , 95% confidence intervals, Welch’s p -values, and Cohen’s d effect sizes are provided.

Comparison	Mean difference (Δ)	95% CI of Δ	t (Welch)	p -value	Cohen’s d	Interpretation
SD = 0.10 vs SD = 0.05	12.24	(5.76, 18.72)	3.73	$2.6\times 10^{-4}$	0.53	Significant, medium effect
SD = 0.10 vs SD = 0.01	12.54	(6.12, 18.96)	3.86	$1.6\times 10^{-4}$	0.55	Significant, medium effect
SD = 0.05 vs SD = 0.01	0.30	(−4.62, 5.22)	0.12	0.90	0.02	Not significant, negligible effect

**TABLE 13:** Pairwise comparisons of the DDQN-N algorithm under different sensor noise levels. Mean differences (Δ) , 90%, 95%, and 99% confidence intervals, Welch’s p -values, Cohen’s d effect sizes, and interpretation are provided.

Comparison	Δ	90% CI	95% CI	99% CI	p -value	Cohen’s d	Interpretation
SD = 0.10 vs SD = 0.05	4.33	(1.02, 7.64)	(0.39, 8.27)	(−0.87, 9.53)	0.032	0.31	Significant at 95%, small effect
SD = 0.10 vs SD = 0.01	4.56	(1.61, 7.51)	(1.04, 8.08)	(−0.09, 9.21)	0.011	0.36	Significant at 95%, small–medium effect
SD = 0.05 vs SD = 0.01	0.23	(−2.44, 2.90)	(−2.95, 3.41)	(−3.97, 4.43)	0.887	0.02	Not significant, negligible effect

**TABLE 14:** Performance with 90%, 95%, and 99% confidence intervals of the number of trials across algorithms against joint constraints. h denotes the half-width of the CI.

Algorithm	90%		95%		99%	
	Mean ±h	CI	Mean ±h	CI	Mean ±h	CI
DQN, [π/6,π/3]	23.96±4.32	(19.64, 28.28)	23.96±5.16	(18.8, 29.12)	23.96±6.83	(17.13, 30.79)
DQN, [π/3,π/2]	15.77±3.35	(12.42, 19.12)	15.77±4.0	(11.77, 19.77)	15.77±5.3	(10.47, 21.07)
DDQN-N, [π/6,π/3]	12.61±1.89	(10.72, 14.5)	12.61±2.26	(10.35, 14.87)	12.61±2.99	(9.62, 15.6)
DDQN-N, [π/3,π/2]	10.74±1.64	(9.1, 12.38)	10.74±1.96	(8.78, 12.7)	10.74±2.59	(8.15, 13.33)

**TABLE 15:** Pairwise comparison of DDQN-N and DQN algorithms under different joint ranges. Mean difference (Δ) , 90%, 95%, and 99% confidence intervals, Welch’s p -value, Cohen’s d , and interpretation are provided.

Comparison	Δ	90% CI	95% CI	99% CI	p -value	Cohen’s d	Interpretation
DDQN-N [30°–60°] vs [60°–90°]	1.87	(−0.62, 4.36)	(−1.10, 4.84)	(−2.05, 5.79)	0.217	0.18	Not significant, small effect
DQN [30°–60°] vs [60°–90°]	8.19	(2.75, 13.63)	(1.70, 14.68)	(−0.38, 16.76)	0.014	0.35	Significant at 95%, small–medium effect

**TABLE 16:** Performance with 90%, 95%, and 99% confidence intervals of the number of trials across the DQN-MHA algorithm with different time windows. h denotes the half-width of the CI.

Algorithm	90%		95%		99%	
	Mean ±h	CI	Mean ±h	CI	Mean ±h	CI
DQN-MHA, time window = 4	17.51±3.73	(13.78, 21.24)	17.51±4.45	(13.06, 21.96)	17.51±5.89	(11.62, 23.4)
DQN-MHA, time window = 8	16.01±1.8	(14.21, 17.81)	16.01±2.15	(13.86, 18.16)	16.01±2.84	(13.17, 18.85)
DQN-MHA, time window = 16	15.08±1.62	(13.46, 16.7)	15.08±1.94	(13.14, 17.02)	15.08±2.57	(12.51, 17.65)
DQN-MHA, time window = 32	9.25±2.4	(6.85, 11.65)	9.25±2.86	(6.39, 12.11)	9.25±3.79	(5.46, 13.04)

**TABLE 17:** Pairwise comparisons of the DQN-MHA algorithm under different time windows (TW = 4, 8, 16, 32). Mean differences (Δ) , 90%, 95%, and 99% confidence intervals, Welch’s p -values, Cohen’s d effect sizes, and interpretation are provided.

Comparison	Δ	90% CI	95% CI	99% CI	p -value	Cohen’s d	Interpretation
DQN-MHA (TW = 4) − (TW = 8)	1.50	(−2.62, 5.62)	(−3.42, 6.42)	(−5.00, 8.00)	0.548	0.09	Not significant, negligible effect
DQN-MHA (TW = 4) − (TW = 16)	2.43	(−1.62, 6.48)	(−2.41, 7.27)	(−3.96, 8.82)	0.323	0.14	Not significant, negligible effect
DQN-MHA (TW = 4) − (TW = 32)	8.26	(3.85, 12.67)	(2.99, 13.53)	(1.31, 15.21)	0.002	0.44	Significant at all levels, medium effect
DQN-MHA (TW = 8) − (TW = 16)	0.93	(−1.48, 3.34)	(−1.94, 3.80)	(−2.86, 4.72)	0.524	0.09	Not significant, negligible effect
DQN-MHA (TW = 8) − (TW = 32)	6.76	(3.78, 9.74)	(3.20, 10.32)	(2.07, 11.45)	0.0002	0.53	Significant at all levels, medium effect
DQN-MHA (TW = 16) − (TW = 32)	5.83	(2.95, 8.71)	(2.39, 9.27)	(1.29, 10.37)	0.001	0.47	Significant at all levels, medium effect

### 4.4 Limitations

Although the proposed architectures show good results in throwing tasks, they have several important limitations. First, using discrete action spaces constrains the task’s performance. Discretizing joint angles makes learning more manageable but reduces precision, which can be problematic when highly accurate throwing is required.

Second, the methods depend on manually designed reward functions. These rewards were determined through trial and error and may not generalize effectively to other robots or tasks. The speed of learning and how quickly the model converges also depend heavily on how the state and reward functions are designed.

Third, although attention-based models such as DQN-MHA improve performance, they incur additional computational costs. Their complexity can limit real-time use on robots, particularly in resource-constrained environments. Finally, the models were trained in simplified environments. Their generalization to dynamic, real-world settings with more complex physics remains to be demonstrated.

## 5 Conclusion and future work

This paper serves multiple purposes: it provides a detailed review of state-of-the-art deep reinforcement learning algorithms for robotic throwing and introduces two novel approaches to enhance deep Q-networks by integrating self-attention mechanisms. The proposed models demonstrate better performance than the standard DQN across all experiments. Although the new models outperform other algorithms in specific situations, they exhibit limitations in others. Notably, DQN with multi-head attention outperforms DQN with structured self-attention. The latter’s complexity and its reliance on attention heads reduce its effectiveness for the throwing problem compared to the multi-head attention approach.

Combining various techniques with DQNs could prove valuable for more complex robotic applications. Transformer architectures may significantly improve the precision and adaptability of robotic manipulators performing throwing tasks. Advanced transformer mechanisms, such as multi-head attention, enable simultaneous consideration of critical factors, including object trajectory and manipulator positioning, thereby enhancing accuracy and efficiency.

Future work will focus on tackling throwing tasks with reinforcement learning algorithms designed for continuous action spaces, such as policy gradient and actor-critic methods, to improve performance under real-world constraints.

We also plan to test the proposed architectures in higher-fidelity simulators such as NVIDIA Isaac Sim, which will allow us to incorporate more realistic physics and dynamic scenarios. Additionally, we will explore transfer learning techniques to apply knowledge from one robotic system to related tasks, such as picking and placing, object catching, handing off, and sorting.

Although this work focused on evaluating the overall performance of different DQN-based architectures in a robotic throwing scenario, future investigations will include ablation studies to better understand the contribution of individual components such as attention mechanisms or prioritized replay buffers. Such an analysis would provide additional insights into the interpretability of the learned policies. Furthermore, statistical hypothesis testing, such as Welch’s test, can be beneficial in more marginal cases and will be considered in future analyses.

## Data Availability

The original contributions presented in the study are included in the article/supplementary material; further inquiries can be directed to the corresponding author.

## Footnotes

1 The list of acronyms used in this paper is provided in Table 1.

2
https://github.com/MhdNur9/DQN_Types
